# The speciation and adaptation of the polyploids: a case study of the Chinese *Isoetes* L. diploid-polyploid complex

**DOI:** 10.1186/s12862-020-01687-4

**Published:** 2020-09-14

**Authors:** Xiaokang Dai, Xiang Li, Yuqian Huang, Xing Liu

**Affiliations:** 1 Laboratory of Plant Systematics and Evolutionary Biology, College of Life Sciences, Wuhan University, Wuhan, 430072 Hubei People’s Republic of China; 2 College of Sciences, Tibet University, Lhasa, 850012 Tibet People’s Republic of China

**Keywords:** Allopolyploid, Altitude, Chinese *Isoetes*, Distribute pattern, Niche breadth, Niche novelty

## Abstract

**Background:**

The Chinese *Isoetes* L. are distributed in a stairway pattern: diploids in the high altitude and polyploids in the low altitude. The allopolyploid *I. sinensis* and its diploid parents *I. yunguiensis* and *I. taiwanensis* is an ideal system with which to investigate the relationships between polyploid speciation and the ecological niches preferences.

**Results:**

There were two major clades in the nuclear phylogenetic tree, all of the populations of polyploid were simultaneously located in both clades. The chloroplast phylogenetic tree included two clades with different populations of the polyploid clustered with the diploids separately: *I. yunguiensis* with partial populations of the *I. sinensis* and *I. taiwanensis* with the rest populations of the *I. sinensis*. The crow node of the *I. sinensis* allopolyploid system was 4.43 Ma (95% HPD: 2.77–6.97 Ma). The divergence time between *I. sinensis* and *I. taiwanensis* was estimated to 0.65 Ma (95% HPD: 0.26–1.91 Ma). The narrower niche breadth in *I.sinensis* than those of its diploid progenitors and less niche overlap in the pairwise comparisons between the polyploid and its progenitors.

**Conclusions:**

Our results elucidate that *I. yunguinensis* and *I. taiwanensis* contribute to the speciation of *I. sinensis*, the diploid parents are the female parents of different populations. The change of altitude might have played an important role in allopolyploid speciation and the pattern of distribution of *I. sinensis*. Additionally, niche novelty of the allopolyploid population of *I. sinensis* has been detected, in accordance with the hypothesis that niche shift between the polyploids and its diploid progenitors is important for the establishment and persistence of the polyploids.

## Background

Polyploidy, or whole-genome duplication (WGD), is widespread in plants, fungi and animals [1–3]. Allopolyploidy and autopolyploidy are the two ways to duplicate chromosomal materials: the former one originates from a hybridization of two species with associated genome duplication while the latter one results from genome duplication within one species [4–6]. WGD is one of the most important forces for vascular plant evolution [7]. Nearly 25% of vascular plants are recent polyploids [7], and more than 15% of angiosperm species and 30% of ferns are estimated to be polyploids through speciation [8]. Additionally, an ancient WGD event occurring in flowering plants is hypothesized to have catalysed key innovations that led to the success and diversification of major clades of angiosperms [9]. Polyploidy has extensive genetic, physiological, morphological, and ecological ramifications [10]. The increasing genome size and genomic content which potentially provides strong tolerance to environmental stresses and increases the possibility to colonize new habitats [11–13]. Therefore, the distribution of polyploids is assumed to be more likely in high altitude regions and the ecological niches of polyploids should be both distinct and broader than those of their diploid progenitors [14, 15].

*Isoetes* L. is an ancient heterosporous Lycopsids with worldwide distribution and there are approximately 150–350 extant species [16]. The origin of *Isoetes* can be dated back to the Devonian Period and this genus is one of the representatives of early diverging plants [17, 18]. Six *Isoetes* species are reported in China: *I. hypsophila* [19], *I. shangrilaensis* [20], *I. yunguiensis* [21], *I. taiwanensis* [22], *I. sinensis* [23] and *I. orientalis* [24]. However, the distribution of *Isoetes* in China doesn’t adhere to the hypothesis that the distribution of polyploids is assumed to be more likely in high altitude regions. They are distributed in a stairway pattern: parts of diploids in the high elevation: *I. hypsophila* and *I. shangrilaensis* in the Qinghai-Tibet Plateau (QTP), the first step, and *I. yunguiensis* in the Yunnan-Guizhou Plateau (YGP), the second step; polyploids and one diploid in the low elevation: the polyploids *I. sinensis* and *I. orientalis* in the middle and lower reaches of the Yangtze River, and the diploid *I. taiwanensis* in the Taiwan and Kinmen Islands, the third step.

The morphological evidence, intermediate megaspore texture of *I. sinensis* between those of *I. yunguiensis* and *I. taiwanensis*, suggests that these two diploid *Isoetes* are the parents of *I. sinensis* [25–27]. In addition, Taylor’s research supported this hypothesis because two different clones of the second intron of a *LEAFY* homolog were detected from *I. sinensis*: one of them similar to the *I.taiwanensis* sequence while the other similar to *the I. yunguiensis* sequence [25]. The allopolyploid *I. sinensis* and its diploid parents (*I. yunguiensis* and *I. taiwanensis*) is an ideal system with which to investigate the relationships between polyploid speciation and changes in ecological preferences. Therefore, we would like to examine whether this distribution pattern corresponds to any niche shift among the polyploid and its progenitors, which can provide evidence toward the reason for the establishment and persistence of the polyploid. In this research, we quantified and compared the ecological niche shifts using ecological niche models in geographic space [28, 29] and using multivariate statistics in environmental space [30, 31].

The change of altitude might have played an important role in allopolyploid speciation and the pattern of distribution of the genus *Isoetes* of China [32]. By calculating the divergence time among species, we can associate the distinct pattern of distribution with tectonic. Larsen [33] and Kim [34] used several chloroplast locus and nr*ITS* to estimate the divergence time of the *Isoetes* in worldwide. However, there is a discrepancy in the divergence time of two diploid species *I. yunguiensis* and *I. taiwanensis*. Larsen suggested that the divergence time in East Asian *Isoetes* was 5 Ma [33], while the divergence time between *yunguiensis* and *I. taiwanensis* was 11.1 Ma in the Kim’s research [34]. Several molecular marker can only provide limited genetic information and therefore may have conflicts in different research. In order to verify whether the distribution pattern is related to the tectonic of China, thirteen chloroplast genome of *Isoetes* is used to calculate the divergence among the allopolyploid *I. sinensis* and its diploid parents *I. yunguiensis* and *I. taiwanensis*.

Although previous researches proved that the allopolyploidization of *I. sinensis*, the maternal donor of *I. sinensis* still not clearly explained. The chloroplast phylogenetic tree can provide the information for the maternal origin of allopolyploid populations of *I. sinensis* [25, 35]. Kim used one chloroplast DNA *trnS-psbC* spacer regions to infer that *I. yunguiensis* was likely the maternal genome donor in the allopolyploidization process of *I. sinensis* [35]. We think that only one chloroplast spacer regions and one individual cannot fully explain the maternal origin of *I. sinensis*. In the present research, we enlarged the sample size to population level and used four plastid genetic markers (*ycf66*, *atpB-rbcL*, *petL-psbE* and *trnS-trnG*) to speculate the maternal origin of *I. sinensis*. Meanwhile, we used the second intron of *LEAFY* to verify the hybridization of *I. sinensis* in the population level. Additionally, another polyploid *I. orientalis* has never been included in previous analysis. In the present study, we also clarify the origins of *I. orientalis* by examining both nuclear and plastic sequences.

To investigate the relationship between species distribution patterns and the tectonic of China, we estimated the divergence time among the *I. sinensis* allopolyploid system. We also discuss the maternal origin and hybridization of *I. sinensis* in population level. In this study, we used the environmental factor at the China scale (temperature, precipitation) to define and compare the realized climatic niches of the diploid (*I. yunguiensis* and *I. taiwanensis*) and the tetraploid (*I. sinensis*).

## Results

### Nuclear DNA

The length of the aligned *LEAFY* sequence was 1017 bp. Two hundred ninety-two haplotypes were identified from 380 *LEAFY* sequences. Most shared haplotypes were detected within species, such as H2 and H108 in *I. yunguiensis*, H72 in *I. taiwanensis*, H92 in *I. sinensis* and H133 in *I. orientalis* (Table S3). And one shared haplotype (H51) was found between JD1 and JM from two different species, *I. sinensis* and *I. taiwanensis* (Fig. 2a, Table S3).

There were two major clades in the nuclear phylogenetic tree (Fig. 3). The diploids were exclusively found in either of these clades: *I. yunguiensis* in Clade I and *I. taiwanensis* in Clade II*.* Most populations of *I. sinensis* were simultaneously located in both clades, for example, JD (JD1 and JD2), XN, NX, and HT. Two populations from *I. sinensis*, TD and TT were only found in Clade II*.* And all population of *I. orientalis* were also located in both clades.

### Multiple maternal lineages of *Isoetes*

The lengths of the aligned sequences of *ycf66*, *atpB-rbcL*, *petL-psbE* and *trnS-trnG* were 493, 813, 1428 and 836 bp respectively and the concatenated sequences were 3570 bp. Two major clades were inferred in the plastid phylogenetic tree. Clade A was composed of all the populations from *I. yunguiensis* and the polyploid populations of HT and JD. Clade B consisted of all the populations from *I. taiwanensis*, and the polyploidy populations of XN, TT, NX, TD, and SY (Fig. 4). And two shared haplotypes were found from different species, *I. sinensis* and *I. orientalis*, H21 for populations TD and SY, H24 for populations TD, TT, and SY (Fig. 2b).

### Analysis of divergence times

The BEAST dating analysis estimated the crow node of the *I. sinensis* allopolyploid system was 4.43 Ma (95% HPD: 2.77–6.97 Ma) falling into later Miocene to early Pliocene (Fig. 5). The divergence time between *I. sinensis* and *I. taiwanensis* was estimated to 0.65 Ma (95% HPD: 0.26–1.91 Ma) around the Pleistocene of Quaternary (Fig. 5).

### Niche variation and quantification in geographical space

**Fig. 1:**
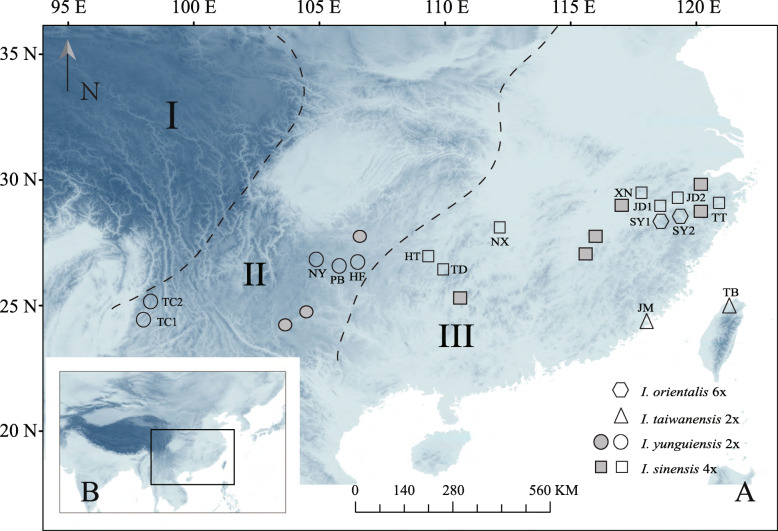
The map was download from WorldClim 1.4 (www.worldclim.org), and it is licensed under a Creative Commons Attribution-ShareAlike 4.0 International License (http://creativecommons.org/licenses/by-sa/4.0/). Geographical distributions of the sampled populations of Chinese *Isoetes* complex: hexagons, triangles, circles and squares are used to represent *I. orientalis*, *I. taiwanensis*, *I. yunguiensis* and *I. sinensis* respectively. The populations colored as grey are extinct. The dotted lines delimit the three distinct elevation stairs (elevation decreases from left to right) in China

The ENMs for *I. sinensis* and its diploid progenitors showed good performances based on their high AUC values (greater than 0.9 for all models). The predicted current distributions of these species were consistent with their present distributions (Fig. 1 and Fig. 6a, b, c). The niche breadths for *I. yunguiensis*, *I. taiwanensis* and *I. sinensis* were 0.25, 0.014 and 0.008 respectively (Fig. 6d). The Schoener’s D index between *I. sinensis* and *I. yunguiensis* was 0.08, 0.26 between *I. sinensis* and *I. taiwanensis*, and 0.17 between *I. yunguiensis* and *I. taiwanensis* (Fig. 6e).

### Niche variation and quantification in ecological space

The first two principal components (PCs) identified by PCA collectively explained 98.1% of the total variation among the three species (PC1 = 74.1%, PC2 = 24%) and clearly separated these species (Fig. 7). Altitude was strongly associated with PC1 and separated *I. yunguiensis* from the others along. Annual precipitation showed a high correlation with PC2 and separated *I. taiwanensis* from the others along. The values of the six retained Bioclim layers of *I. yunguiensis*, *I. taiwanensis* and *I. sinensis* were significantly different (*P* ≤ 0.05) from each other in four out of the six individual environmental variables (Table 1). The ecology of the polyploid species *I. sinensis* were characterized by the highest values for temperature annual range (BIO7) and the lowest values for altitude, with intermediate values for annual mean temperature (BIO1) and annual precipitation (Fig. 8). The ecology of the diploid species *I. taiwanensis* were characterized by the highest values for annual mean temperature, annual precipitation and the lowest values for temperature annual range (Fig. 8). Conversely, the ecology of the diploid species *I. yunguiensis* showed the lowest values for annual mean temperature and annual precipitation but the highest values for altitude (Fig. 8).

**Table 1:** The results of the nonparametric Kruskal test for species pairwise comparison in each climate variable. The asterisk indicates a significant difference between the two species in the respective variable (see also Fig. 7)

climatic variable	*I.sinensis* vs *I.yunguiensis*	*I.sinensis* vs *I.taiwanensis*	*I.taiwanensis* vs *I.yunguiensis*
alt	0.004483(**)	0.004209(**)	0.004209(**)
bio1	0.004414(**)	0.01471(*)	0.004209(**)
bio3	0.004009(**)	0.006876(**)	0.452(n.s.)
bio7	0.004414(**)	0.00801(**)	0.004209(**)
bio12	0.01183(*)	0.008208(**)	0.004209(**)
bio15	0.004483(**)	0.2031(n.s.)	0.004209(**)

** *P* ≤ 0.01

* *P* ≤ 0.05

n.s. *P* > 0.05

## Discussion

### Hybridization and polyploidization complicate the speciation of the Chinese *Isoetes*

**Fig. 2:**
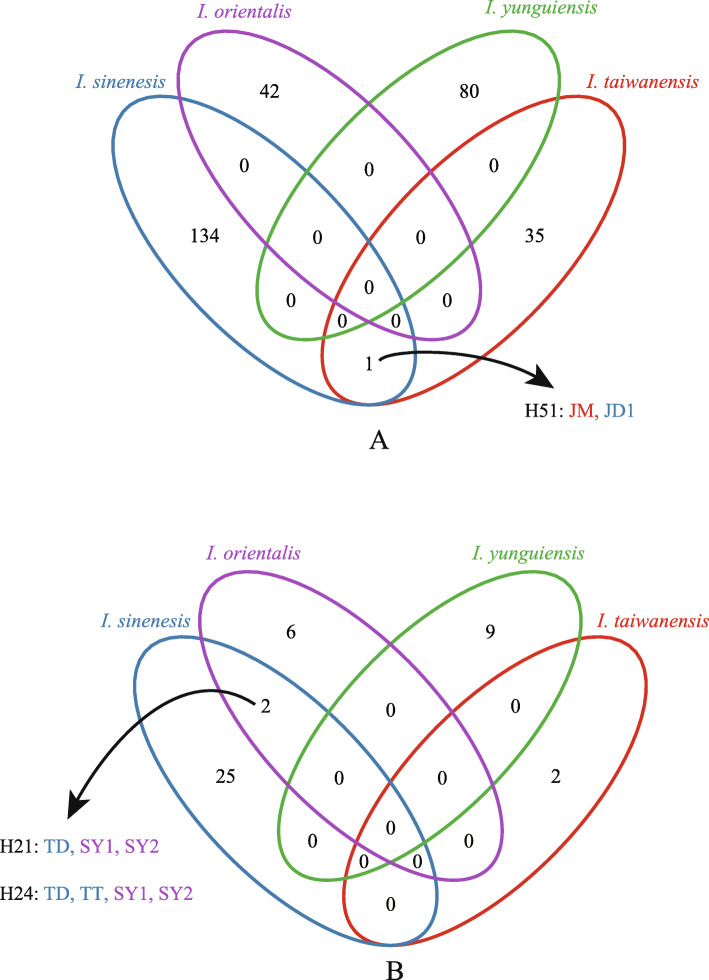
**a**: The Venn diagram of the haplotypes of nuclear DNA data; **b**: The venn diagram of the haplotypes of chloroplast DNA data

**Fig. 3:**
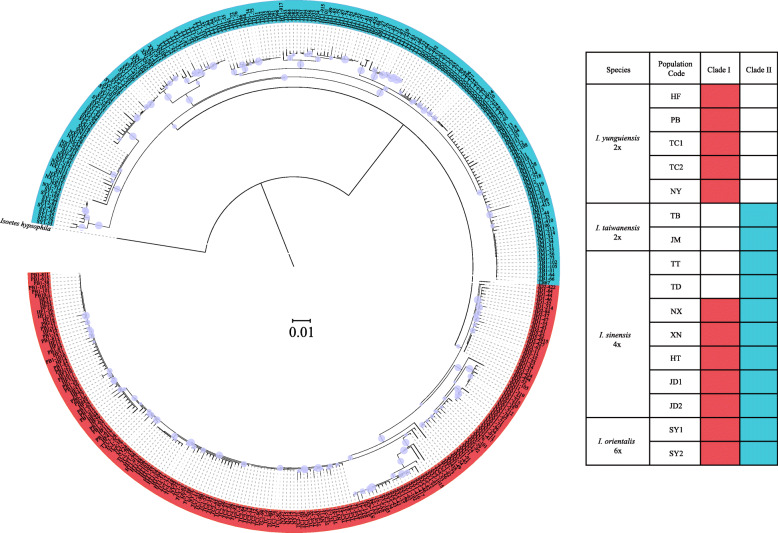
Phylogenetic tree of Chinese *Isoetes* complex based on the second intron of *LEAFY* homologue. The circles on each node represent the strong supports (higher than 0.75). Two clades are denoted by different color bars. The table indicates where the clones of each population are distributed in the phylogenetic tree

Our results verified that most populations of *I. sinensis* originate from the allopolyploidization between *I. yunguiensis* and *I. taiwanensis*. Interestingly, all the clones from the populations TT and TD of *I. sinensis* are exclusively placed in Clade II (Fig. 2), which hints the individuals from these populations might be homozygote. These two populations are in distant geographic locations (Fig. 1) and in different subclades in the nuclear tree (Fig. 3). It would be worthwhile to perform more comparisons between them and other allopolyploid populations in the perspectives of morphology, ecology and so on.

**Fig. 4:**
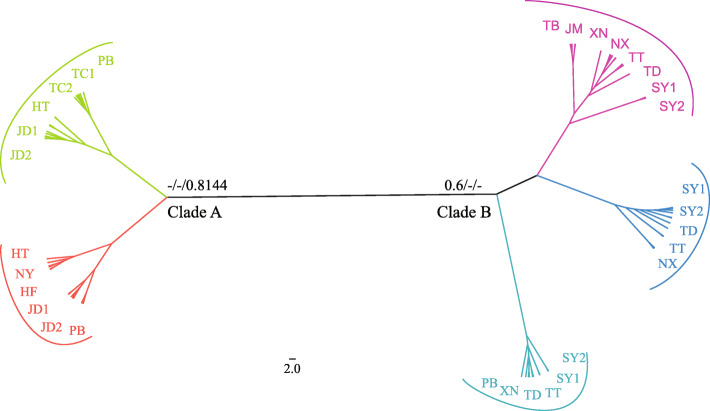
Phylogenic tree of the Chinese *Isoetes* complex based on the four plastid DNA regions including *ycf66*, *atpB-rbcL*, *petL-psbE* and *trnS-trnG* (the posterior probability of Bayesian Inference of Mrbayes/the bootstrap values of Maximum Parsimony analysis/ Posterior possibility of Bayesian Inference from Beast are showed on each clades). Five subclades are denoted by different color bars and the population codes are labeled in each subclade

On the other hand, the chloroplast phylogenetic tree can provide the information for the maternal origin of allopolyploid populations of *I. sinensis* [25, 35]. Kim inferred the diploid *I. yunguiensis* as the maternal progenitor of *I. sinensis* [35]. However, our chloroplast phylogeny confirmed *I. yunguiensis* is the maternal progenitor solely for HT and JD populations whereas the rest populations (TT, TD, XN and NX) showed *I. taiwanensis* as their maternal progenitor (Fig. 4). The main finding here is that *I. sinensis* originated multiple times from reciprocal maternal progenitors, whereas it has previously been suggested a single maternal origin. We originally assumed that the exclusive hybridization pattern might adapt the allopolyploids to the specific environments. Nevertheless, we do not detect any ecological niche differences in ecological space between the populations with the different maternal contributors (Fig. S1, Table S6). Thus, this exclusive hybridization pattern possibly results from the founder effect, although further investigations is needed to validate this hypothesis.

In addition, *I. orientalis* (hexaploid) was found by Liu et al. in China, Songyang County of Zhejiang Province in 2006 [24]. But the speciation of *I. orientalis* has never been explored. In this study, we discuss the hybridization and polyploidization of *I. orientalis* in the first time. In the nuclear tree, the clones from each *I. orientalis* individuals separated in both two major clades means that both Yungui and Taiwan ancestral lineages may be involved in the speciation of this species (Fig. 3). Additionally, two shared chloroplast haplotypes are detected between the homozygote populations of *I. sinensis* and *I. orientalis* (Fig. 2b) and these populations also clustered with *I. orientalis* in the same clade of the chloroplast tree (Fig. 4). We hypothesize that *I. orientalis* is possibly formed by the hybridization between the *I. yunguinensis* (paternal donor) and the homozygote (the populations of TT and TD) from *I. sinensis* (maternal donor). Similar scenarios are observed in other hexaploids *Isoetes*, *Isoetes* japonica and *Isoetes* coreana. *I. coreana* originates from the diploid *I. taiwanensis* and the tetraploid *Isoetes hallasanensis*, while *I. japonica* comes from *I. taiwanensis* and *I. sinensis* [35].

### *I. sinensis* allopolyploid system distribute pattern and the uplifted of land

**Fig. 5:**
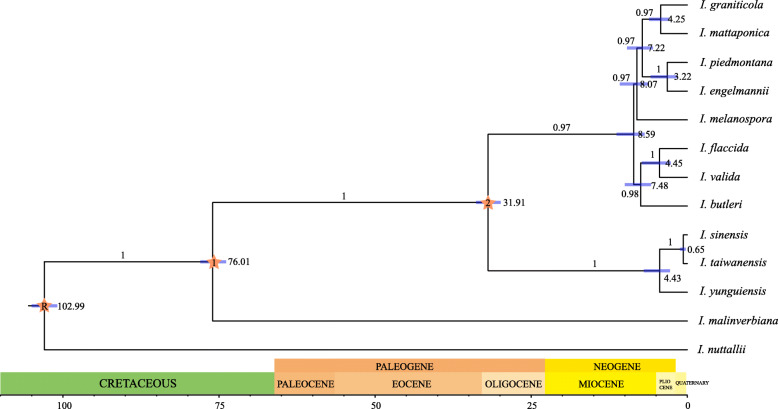
Chronogram showing divergence times estimated in BEAST based on chloroplast genome data. Blue bars represent 95% high posterior density for the estimated mean dates. Node labeled R, 1 and 2 is the calibration point (for more details, see Materials and Methods)

Previous study hypothesized that the polyploidy speciations of *Isoetes* in East Asia might originate and develop from Holocene (Quaternary) and occurring only in low altitude regions [32]. But this hypothesis did not based on the divergence time among these species. The age estimate for the node *I. sinensis* allopolyploid system is 4.43 ma, when the *I. yunguiensis* lineage is distinct to the *I. taiwanensis* lineage (*I. taiwanensis* and *I. sinensis*, Fig. 5). The chloroplast genome sample of *I. sinensis* was acquired from Fuzhou City (119°23′89.30″E, 26°08′76.41″N), Fujian Province of China [36]. We assume that *I. taiwanensis* is the maternal progenitor for this population of *I. sinensis*. And the divergence time of this lineage is 0.65 Ma (Fig. 5).

Hybrid speciation is a form of speciation where hybridization between two different species, hence the species splits between two parents is the first step of hybrid. Therefore, the allopolyploidization of *I. sinensis* might happen during 4.43 Ma (Pliocene) to 0.65 Ma (Pleistocene). The YGP has consistently responded to each period of the Qinghai–Tibetan Plateau’s uplifting, which reached its present elevation some 3.4 Ma, during the Pliocene [37]. The allopolyploidy speciations of *I. sinensis* may originate and develop from Pliocene to Pleistocene, after the uplifting of the YGP. Our results consist with part of the hypothesis: the allopolyploidization of *I. sinensis* only occurred in low altitude regions but earlier than Holocene (Quaternary). Dispersal of *Isoetes* spores is often accomplished via floating leaves [38], which is impossible from low to the high elevation. Therefore, polyploids with better adaptability are only found in the low altitude region. Our results conclude that the change of altitude might have played an important role in allopolyploid speciation and the pattern of distribution of the Chinese *Isoetes*.

### Niche variation and quantification in geographical and ecological space

**Fig. 6:**
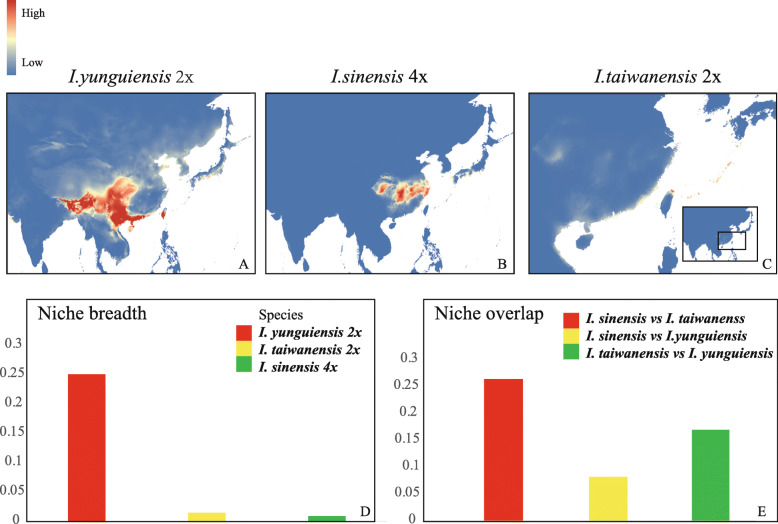
**a-c**: The projected distributions for the allopolyploid (*I. sinensis*) and the diploid parents (*I. taiwanensis* and *I. yunguiensis*) under the current climate conditions. The color of the area indicates the occurrence probability (red means high probability while blue means low probability). **d**: The niche breadth of the allopolyploid (*I. sinensis*) and the diploid parents (*I. taiwanensis* and *I. yunguiensis*). **e**: The niche overlap between each species

Polyploids, characterized by increased genome size and genomic content, are expected to have higher ecological tolerances and broader ecological niche breadth than their diploid progenitors [12, 13]. However, the difference of the niche breadth between the polyploids and diploids can vary in different situations. For instance, there is no significant difference of niche breadth within *Claytonia perfoliata* species complex [39], broader niche breadth in *Clarkia* polyploids [40], and narrower niche breadth in *Primul*a polyploids [15]. The highest niche breadth in our *Isoetes* allopolyploid system is the breadth of *I. yunguinensis*, 0.25 while the breadths of *I. sinensis* and *I. taiwanensis* are much narrower and similar, 0.014 and 0.008 respectively (Fig. 6d). Instead of *I. sinensis*, *I. yunguinensis* fits in with the expectation for polyploids that inhabit to harsher environments (higher altitude regions) and possess broader niche breadth. This might be related to the older age and better adaptation of *I. yunguiensis* before the origin of the polyploids, which is consistent with the similar situation in *Primula*. *Primula farinosa* can spread and adapt to various environments before the origin of the polyploid species because of its older age and higher levels of genetic morpholisms [15]. It will be worthwhile to explore the underlying genetic mechanisms for such ecological consequences.

**Fig. 7:**
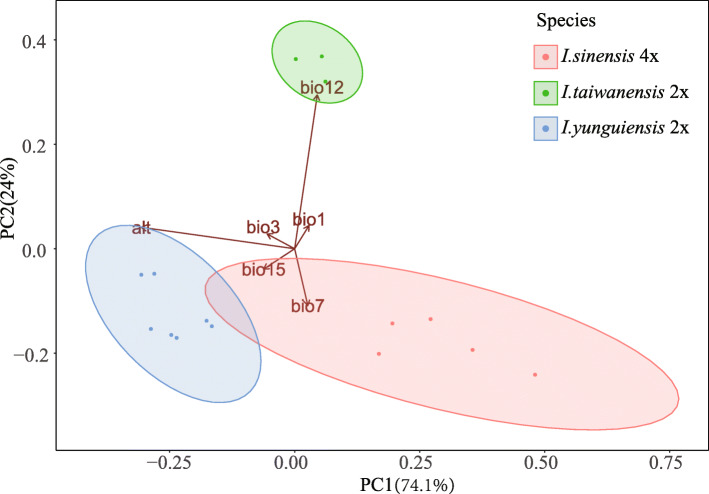
Principal component analysis for environmental variables of the allopolyploid (*I. sinensis*) and the diploid parents (*I. taiwanensis* and *I. yunguiensis*). The percentage variations explain by each principal component are indicated in parenthesis

**Fig. 8:**
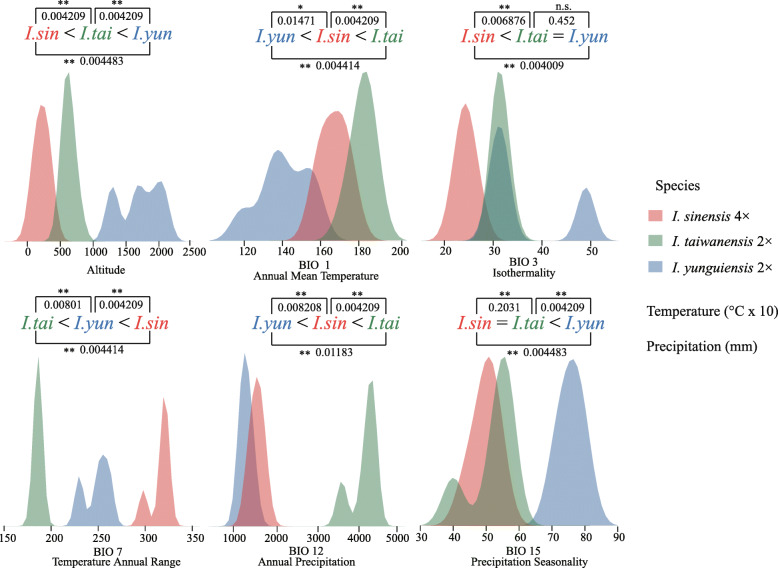
Kernel density plots of the six environmental variables for the *Isoetes* allopolyploid system in China. Differentiation among species and the results of the nonparametric Kruskal-test are indicated in each plot. The equal sign indicates the lack of significant differences (*p* ≥ 0.05), while the significant differences are indicated by either higher or lower sign (*p* < 0.05)

In the terms of niche overlap, *I. sinensis* had Schoener’s D similarity indexes < 0.3 in both pairwise comparisons with its progenitors, which accords with the standards for niche novelty, one pattern of niche shift in Marchant’s research [10]. The PCA in ecological space also clearly separates these three species (Fig. 7). Additionally, the PCA indicates that altitude is one of the primary factors separating the species. This result coincides with the geographical pattern, in which *I. yunguiensis* is distributed at a higher altitude than the other two species (Table 2) [41]. Previous research on *Polystichum saximontanum* obtained the similar conclusion that altitude could have played a significant role in niche shift of the polyploid from its progenitors [10]. Niche differences between allopolyploid and the progenitors are also observed from the kernel density plot (Fig. 8; Table 1). In general, our results agree with the existence of niche shift between the polyploids and its diploid progenitors in our *Isoetes* allopolyploid system. Niche novelty is the approach *I. sinensis* took when it established and persisted, and altitude plays a crucial role in this niche shift.

**Table 2:** Ploidy, population code, number of samples for plastid DNA (NS) and number of clones for nuclear DNA (NC) and locations (including geographical coordinates and altitudes) of the Chiese *Isoetes* complex and one outgroup species (*I. hypsophila*)

	Species	Ploidy	Population code	NS	NC	Location	Geographical Coordinates	Altitude/m
Outgroup	*I. yunguiensis*	2	HF	4	20	Hongfenghu, Guizho, China	N 26° 29′; E 106° 58′	1231
		2	PB	5	25	Pingba, Guizhou, China	N 26° 25′; E 106° 17′	1280
		2	TC1	5	25	Tengchong, Guizhou, China	N 24° 52′; E 098° 34′	1769
		2	TC2	5	25		N 25° 00′; E 098° 34′	2063
		2	NY	2	10	Nayong, Guizhou, China	N 22° 44′; E 105° 23′	1672
	*I. taiwanensis*	2	TB	5	25	Taibei, Taiwan, China	N 25° 10′; E 121° 33′	880
		2	JM	3	25	Kinmen, Taiwan, China	N 24° 27′; E 118° 23′	94
	*I. sinensis*	4	HT	10	25	Huitong, Hunan, China	N 26° 45′; E 109° 37’	284
		4	TD	10	25	Tongdao, Hunan, China	N 26° 20′; E 109° 51′	494
		4	NX	10	25	Ningxiang, Hunan, China	N 28° 11′; E 112° 17’	84
		4	XN	10	25	Xiuning, Anhui, China	N 29° 42′; E 118° 09′	360
		4	TT	10	25	Tiantai, Zhejiang, China	N 29° 15′; E 121° 05’	942
		4	JD1	10	25	Jiande, Zhejiang, China	N 29° 28′; E 119° 14′	134
		4	JD2	10	25		N 29° 28′; E 119° 15′	134
	*I. orientalis*	6	SY1	10	25	Songyang, Zhejiang, China	N 28° 47′; E 119° 12’	235
		6	SY2	10	25		N 28° 46′; E 119° 12’	183
	*I. hypsophila*	2	BY	1	1	Baiyu,Sichuang, China	N 30° 58′; E 099° 37′	3980

## Conclusions

Our results elucidate that *I. yunguinensis* and *I. taiwanensis* contribute to the speciation of *I. sinensis*, while some of them (homozygote) originate from the *I. taiwanensis*. Besides, our chloroplast phylogeny confirmed *I. yunguiensis* is the maternal progenitor solely for HT and JD populations whereas the rest populations (TT, TD, XN and NX) showed *I. taiwanensis* as their maternal progenitor. The interesting and complex hybrid patterns have been detected at the population level. The change of altitude might have played an important role in allopolyploid speciation and the pattern of distribution of *I. sinensis*. The speciation of *I. orientalis* has been first explored in this research. We hypothesize that *I. orientalis* is formed by the hybridization between *I. yunguiensis* as paternal progenitor and the homozygote (the populations of TT and TD) from *I. sinensis* as maternal progenitor. The change of altitude might have played an important role in allopolyploid speciation and the pattern of distribution of the Chinese *Isoetes*. Additionally, niche novelty of *I. sinensis* has been detected which is in accordance with the hypothesis that niche shift between the polyploids and its diploid progenitors is important for the establishment and persistence of the polyploids. Meanwhile, the narrower niche breadth has been found in the polyploid than its diploid progenitors.

## Methods

### Population sampling

One twenty-one individuals from 16 populations were used (including one outgroup). Five to ten plants were sampled in each population (Fig. 1; Table 2). The ploidy level of the plants has been determined by chromosome counts or spore size measurements [26, 27, 32].

### DNA extraction, PCR amplification and sequencing

Total genomic DNA was extracted from 0.5 g silica-dried leaf tissue for each sample through a modified cetyltrimethylammonium bromide (CTAB) procedure [42]. The second intron of *LEAFY* (*LEAFY*) was amplified using the primers 30F and 1190R [25]. After preliminary screening, introns of *ycf66*, the intergenic regions of *atpB-rbcL* [43], *petL-psbE* [44] and *trnS-trnG* [45], were chosen as plastid genetic markers. The primers for *ycf66* and *petL-psbE* were designed according to the complete chloroplast genome of *Isoetes flaccida* [46]. The PCR products of *LEAFY* were purified by an agarose gel DNA extraction kit (BioTeke, China), and then cloned into a pMD19-T vector system (TaKaRa Biotechnology (Dalian) Co., Ltd., China). Five positive clones of *LEAFY* from each accession were selected. All the PCR products and were sequenced in both directions (except for *ycf66*, which was sequenced in one direction because of the short length) on an ABI 3730 DNA Sequencer using BigDye Terminator version 3.1 (Applied Biosystems). The one-directional sequences (*ycf66*) were checked using the software Chromas 1.62 (Technelysium Pty, Australia) and the two complementary sequences were assembled using ContigExpress 6.0.620.0 (InforMax, Inc., USA). All the nuclear and plastid sequences were uploaded to GenBank (the National Center for Biotechnology Information, http://www.ncbi.nlm.nih.gov). The serial numbers of these sequences are detailed in Additional file 1: Table S1 and S2.

### Phylogenetic analyses of nuclear and plastid marker

DnaSP version 5.0.0 was used to identify the haplotypes in the nuclear sequences [47]. Maximum likelihood (ML) was used to construct the nuclear phylogenetic tree. ML trees were constructed by FastTree 2.1.10 using GTR + CAT model and other default settings [48].

The incongruence length difference (ILD) test, was performed in PAUP* 4.0 for the plastid DNA sequences (*ycf66*, *atpB-rbcL*, *petL-psbE* and *trnS-trnG*) using 100 homogeneity replicates with heuristic searches under parsimony analysis [49, 50]. The result of the ILD test indicated that there was no incongruence among the plastid DNA marker (*P* > 0.05). Accordingly, sequences of the four plastid DNA regions were concatenated into one sequence for subsequent analysis. DnaSP version 5.0.0 was used to identify the haplotypes in the plastid sequences [47].

Maximum parsimony (MP) and Bayesian inference (BI) were used to construct the chloroplast phylogenetic tree. The best-fit models HKY + G was chosen for the plastid DNA data, based on the Akaike information criterion (AIC) standards in MrModeltest [51]. MP analysis was performed using the program PAUP* 4.0 [50]. A heuristic searches strategy was employed; 500 replicates with random taxon-addition sequences in combination with tree-bisection-reconnection branch swapping; the option MULPARS functional was kept but not STEEPEST DESCENT. Node support was estimated with 1000 bootstrap values [52]. BI analysis was conducted using the program MrBayes 3.1 [53]. Bayesian Markov chain Monte Carlo (MCMC) inference was used in two independent replicates of four simultaneous chains, with a starting random tree, for 1000,000 MCMC generations, and tree sampled every 1000 generations; first 25% of the 1000,000 generations discarded as a burn-in. The posterior probability was calculated from the consensus of the remaining trees. Moreover, BEAST v 1.8.0 was used to further attest the relationship of plastid sequences with the same nucleotide substitution model (HKY + G) [54]. Two MCMC chains run for 8,000,000 generations with sampling every 1000 generations. Tracer v1.6 (http://beast.bio.ed.ac.uk/Tracer) was used to check whether the values of the parameter effective sample size (ESS) were greater than 200, which ensures a good mixing of MCMC chains. Trees were summarized and annotated using TreeAnnotator v1.8.0, after discarding by the first 25% generations.

### Analysis of divergence times

The chloroplast genome data of *Isoetes* was downloaded from GenBank and the detail accession number was present in Table 3. Sequences were aligned by the program MATTT [55] with the “auto” strategy. The ML chloroplast genome phylogenetic tree was estimated by IQ-TREE [56]. The divergence time of different lineages was estimated by a Bayesian approach implemented in BEAST v 1.8.4 [57]. The tree estimated by IQ-TREE was used as starting tree. The BEAUti interface was used to generate input files for BEAST, in which a GTR + I + G model for the combined dataset was applied with a Yule speciation tree prior and an uncorrelated lognormal molecular clock model. Three calibrated nodes were refer to TimeTree web resource (http://www.timetree.org/) and Larsen’s result [32]. Set a normal distribution on the age of the root, the treeModel.rootHeight parameter and choose normal. Parameterize the normal distribution so that the mean and initial value are equal to 103 and the stdev (standard deviation) is equal to 10. Node 1and 2 was also used the normal distribution with an offset of 76 and 32 respectively, the stdev is both equal to 10. The BEAST trees were sampled every 1000 generations and ran for 10,000,000 generations. Tracer v1.5 (http://beast.bio.ed.ac.uk/Tracer) was used to check whether the values of the parameter effective sample size (ESS) were greater than 200. The final annotation was completed by using TreeAnnotator v1.8.0 after discarding the first 10% generations.

**Table 3:** Chloroplast genome download information

Species	Accession number
*I. butleri*	NC_038071.1
*I. engelmannii*	NC_038080.1
*I. flaccida*	NC_014675.1
*I. graniticola*	NC_039821.1
*I. malinverbiana*	NC_040924.1
*I. mattaponica*	NC_039703.1
*I. melanospora*	NC_038072.1
*I. nuttallii*	NC_038073.1
*I. piedmontana*	NC_040925.1
*I. sinensis*	MN172503.1
*I. taiwanensis*	MF149843.1
*I. valida*	NC_038074.1
*I. yunguiensis*	NC_041146.1

### Niche variation and quantification in geographical space

We defined the allopolyploid population of *I. sinensis* and its diploid progenitors, *I. yunguiensis* and *I. taiwanensis*, as an allopolyploid system. To predict the suitable distributions of all the *Isoetes* in this allopolyploid system under the current climate conditions, eighteen occurrence records were used: five records of *I. sinensis*, seven records of *I. yunguiensis* and six records of *I. taiwanensis* (Table S5). Most of these occurrence records were gathered from georeferenced specimens [41], while four records of *I. taiwanensis* collected from GBIF (https://www.gbif.org/) and Plants of Taiwan (http://tai2.ntu.edu.tw/index.php). We used all 19 environmental layers and altitude information from the WorldClim 1.4 dataset (http://www.worldclim.org/) with the spatial resolution of 2.5 arc-minutes for training and projecting ENM models, and it is licensed under a Creative Commons Attribution-ShareAlike 4.0 International License (http://creativecommons.org/licenses/by-sa/4.0/) [58]. To avoid possible confounding effects of correlated variables and overfitting of niche models, correlation coefficients were calculated using the ‘correlation and summary stats’ tool of SDMtoolbox in ArcMap [10, 59]. Six Bioclim layers (altitude, annual mean temperature, isothermality, temperature annual range, annual precipitation, and precipitation seasonality) were retained based on their correlation values below |0.75|. ENM models were constructed for each species through 10 replicates in MaxEnt 3.3.3 k with 75% of the occurrence records to calibrate the model and 25% to test it [60]. The area under the ‘receiver operating characteristic curve’ (AUC) was used to assess the performance of each model [61]. The average predicted models for each species were employed to calculate the niche breadth and the pairwise niche overlap in geological space by ENMTools1.3 [41, 62]. The Schoener’s D similarity index is the measurement of the niche overlap, with the index ranging from 0 (no niche overlap) to 1 (identical niches) [63].

### Niche variation and quantification in ecological space

The values of the six retained Bioclim layers for each occurrence record were extracted by the ‘Point sampling tool’ plugin in QGIS v2.18.13 (http://qgis.org) and used as inputs to quantify the niche overlap and divergence within the three *Isoetes* in our allopolyploid system through a principal component analysis (PCA). The nonparametric Kruskal–Wallis test was used to compare species for each environmental factor and kernel density plots were used to visualize these differences [64]. All statistical analyses and plots were conducted in R [65].

## Supplementary information

**Additional file 1: Table S1.** The serial numbers of plastid DNA sequences in this study. **Table S2.** The serial numbers of nuclear DNA sequences in this study. **Table S3.** Haplotypes information of nuclear DNA data. **Table S4.** Haplotypes information of cpDNA data. **Table S5.** Location records used for ecological niche modeling. **Table S6.** Results of the nonparametric Kruskal test applied for the populations whose maternal contributor are different in the allopolyploid populations of *I.sinensis*.

**Additional file 2: Figure S1.** Kernel density plots of the six environmental variables for the populations whose maternal contributor are different in the allopolyploid of *I. sinensis*. *I. sinensis* (tai) indicates the maternal contributor of the population is *I. taiwanensis* and *I. sinensis* (yun) means the maternal contributor of the population is *I. yunguiensis*. Differentiation between different populations and the results of the nonparametric Kruskal-test are indicated in each plot. The equal sign indicates the lack of significant differences (*p* ≥ 0.05), while the significant differences are indicated by either higher or lower sign (*p* < 0.05).

## Data Availability

All data generated or analyzed during this study are included in this published article.

## Abbreviations

ENM: Ecological niche models
MP: Maximum parsimony
BI: Bayesian inference
AIC: Akaike information criterion
ESS: Effective sample size
AUC: The area under the ‘receiver operating characteristic curve’
PCA: Principal component analysis
