# Supplementary material for: The speciation and adaptation of the polyploids: a case study of the Chinese Isoetes L. diploid-polyploid complex
Source: BMC Evol Biol. 2020 Sep 14;20:118. doi: 10.1186/s12862-020-01687-4 (PMC7490897; doi:10.1186/s12862-020-01687-4)
Supplement: Supplementary file 1 — Additional file 1: Table S1. The serial numbers of plastid DNA sequences in this study. Table S2. The serial numbers of nuclear DNA sequences in this study. Table S3. Haplotypes information of nuclear DNA data. Table S4. Haplotypes information of cpDNA data. Table S5. Location records used for ecological niche modeling. Table S6. Results of the nonparametric Kruskal test applied for the populations whose maternal contributor are different in the allopolyploid populations of I.sinensis. [file 12862_2020_1687_MOESM1_ESM.zip › Table S1.docx]

Table S1. The serial numbers of plastid DNA sequences in this study.

| Gene name | Species | Sequence name | Serial name |
| --- | --- | --- | --- |
| *atp*B*-rbc*L | *I. yunguiensis* | HF1 | KU553350 |
| *atp*B*-rbc*L | *I. yunguiensis* | HF3 | KU553351 |
| *atp*B*-rbc*L | *I. yunguiensis* | HF4 | KU553352 |
| *atp*B*-rbc*L | *I. yunguiensis* | HF5 | KU553353 |
| *atp*B*-rbc*L | *I. yunguiensis* | NY1 | KU553354 |
| *atp*B*-rbc*L | *I. yunguiensis* | NY2 | KU553355 |
| *atp*B*-rbc*L | *I. yunguiensis* | PB-10 | KU553356 |
| *atp*B*-rbc*L | *I. yunguiensis* | PB-2 | KU553357 |
| *atp*B*-rbc*L | *I. yunguiensis* | PB-3 | KU553358 |
| *atp*B*-rbc*L | *I. yunguiensis* | PB-6 | KU553359 |
| *atp*B*-rbc*L | *I. yunguiensis* | PB-8 | KU553360 |
| *atp*B*-rbc*L | *I. yunguiensis* | TC1-3 | KU553361 |
| *atp*B*-rbc*L | *I. yunguiensis* | TC1-5 | KU553362 |
| *atp*B*-rbc*L | *I. yunguiensis* | TC1-7 | KU553363 |
| *atp*B*-rbc*L | *I. yunguiensis* | TC1-8 | KU553364 |
| *atp*B*-rbc*L | *I. yunguiensis* | TC1-9 | KU553365 |
| *atp*B*-rbc*L | *I. yunguiensis* | TC2-1 | KU553366 |
| *atp*B*-rbc*L | *I. yunguiensis* | TC2-5 | KU553367 |
| *atp*B*-rbc*L | *I. yunguiensis* | TC2-6 | KU553368 |
| *atp*B*-rbc*L | *I. yunguiensis* | TC2-7 | KU553369 |
| *atp*B*-rbc*L | *I. yunguiensis* | TC2-8 | KU553370 |
| *atp*B*-rbc*L | *I. orientensis* | SY1-1 | KU553372 |
| *atp*B*-rbc*L | *I. orientensis* | SY1-10 | KU553373 |
| *atp*B*-rbc*L | *I. orientensis* | SY1-22 | KU553374 |
| *atp*B*-rbc*L | *I. orientensis* | SY1-3 | KU553375 |
| *atp*B*-rbc*L | *I. orientensis* | SY1-4 | KU553376 |
| *atp*B*-rbc*L | *I. orientensis* | SY1-5 | KU553377 |
| *atp*B*-rbc*L | *I. orientensis* | SY1-6 | KU553378 |
| *atp*B*-rbc*L | *I. orientensis* | SY1-7 | KU553379 |
| *atp*B*-rbc*L | *I. orientensis* | SY1-8 | KU553380 |
| *atp*B*-rbc*L | *I. orientensis* | SY1-9 | KU553381 |
| *atp*B*-rbc*L | *I. orientensis* | SY2-10 | KU553382 |
| *atp*B*-rbc*L | *I. orientensis* | SY2-3 | KU553383 |
| *atp*B*-rbc*L | *I. orientensis* | SY2-38 | KU553384 |
| *atp*B*-rbc*L | *I. orientensis* | SY2-4 | KU553385 |
| *atp*B*-rbc*L | *I. orientensis* | SY2-42 | KU553386 |
| *atp*B*-rbc*L | *I. orientensis* | SY2-5 | KU553387 |
| *atp*B*-rbc*L | *I. orientensis* | SY2-6 | KU553388 |
| *atp*B*-rbc*L | *I. orientensis* | SY2-7 | KU553389 |
| *atp*B*-rbc*L | *I. orientensis* | SY2-8 | KU553390 |
| *atp*B*-rbc*L | *I. orientensis* | SY2-9 | KU553391 |
| *atp*B*-rbc*L | *I. taiwanensis* | JM1-2 | KU553392 |
| *atp*B*-rbc*L | *I. taiwanensis* | JM1-5 | KU553393 |
| *atp*B*-rbc*L | *I. taiwanensis* | JM1-7 | KU553394 |
| *atp*B*-rbc*L | *I. taiwanensis* | TB-1 | KU553395 |
| *atp*B*-rbc*L | *I. taiwanensis* | TB-10 | KU553396 |
| *atp*B*-rbc*L | *I. taiwanensis* | TB-3 | KU553397 |
| *atp*B*-rbc*L | *I. taiwanensis* | TB-4 | KU553398 |
| *atp*B*-rbc*L | *I. taiwanensis* | TB-6 | KU553399 |
| *atp*B*-rbc*L | *I. sinensis* | HT10 | KU553400 |
| *atp*B*-rbc*L | *I. sinensis* | HT1-1 | KU553401 |
| *atp*B*-rbc*L | *I. sinensis* | HT1-2 | KU553402 |
| *atp*B*-rbc*L | *I. sinensis* | HT1-3 | KU553403 |
| *atp*B*-rbc*L | *I. sinensis* | HT1-4 | KU553404 |
| *atp*B*-rbc*L | *I. sinensis* | HT1-5 | KU553405 |
| *atp*B*-rbc*L | *I. sinensis* | HT1-6 | KU553406 |
| *atp*B*-rbc*L | *I. sinensis* | HT1-7 | KU553407 |
| *atp*B*-rbc*L | *I. sinensis* | HT1-8 | KU553408 |
| *atp*B*-rbc*L | *I. sinensis* | HT1-9 | KU553409 |
| *atp*B*-rbc*L | *I. sinensis* | JD1-1 | KU553410 |
| *atp*B*-rbc*L | *I. sinensis* | JD1-10 | KU553411 |
| *atp*B*-rbc*L | *I. sinensis* | JD1-2 | KU553412 |
| *atp*B*-rbc*L | *I. sinensis* | JD1-3 | KU553413 |
| *atp*B*-rbc*L | *I. sinensis* | JD1-4 | KU553414 |
| *atp*B*-rbc*L | *I. sinensis* | JD1-5 | KU553415 |
| *atp*B*-rbc*L | *I. sinensis* | JD1-6 | KU553416 |
| *atp*B*-rbc*L | *I. sinensis* | JD1-7 | KU553417 |
| *atp*B*-rbc*L | *I. sinensis* | JD1-8 | KU553418 |
| *atp*B*-rbc*L | *I. sinensis* | JD1-9 | KU553419 |
| *atp*B*-rbc*L | *I. sinensis* | JD2-1 | KU553420 |
| *atp*B*-rbc*L | *I. sinensis* | JD2-3 | KU553421 |
| *atp*B*-rbc*L | *I. sinensis* | JD2-4 | KU553422 |
| *atp*B*-rbc*L | *I. sinensis* | JD2-42 | KU553423 |
| *atp*B*-rbc*L | *I. sinensis* | JD2-49 | KU553424 |
| *atp*B*-rbc*L | *I. sinensis* | JD2-5 | KU553425 |
| *atp*B*-rbc*L | *I. sinensis* | JD2-6 | KU553426 |
| *atp*B*-rbc*L | *I. sinensis* | JD2-7 | KU553427 |
| *atp*B*-rbc*L | *I. sinensis* | JD2-8 | KU553428 |
| *atp*B*-rbc*L | *I. sinensis* | JD2-9 | KU553429 |
| *atp*B*-rbc*L | *I. sinensis* | NX1-1 | KU553430 |
| *atp*B*-rbc*L | *I. sinensis* | NX1-10 | KU553431 |
| *atp*B*-rbc*L | *I. sinensis* | NX1-2 | KU553432 |
| *atp*B*-rbc*L | *I. sinensis* | NX1-3 | KU553433 |
| *atp*B*-rbc*L | *I. sinensis* | NX1-4 | KU553434 |
| *atp*B*-rbc*L | *I. sinensis* | NX1-5 | KU553435 |
| *atp*B*-rbc*L | *I. sinensis* | NX1-6 | KU553436 |
| *atp*B*-rbc*L | *I. sinensis* | NX1-7 | KU553437 |
| *atp*B*-rbc*L | *I. sinensis* | NX1-8 | KU553438 |
| *atp*B*-rbc*L | *I. sinensis* | NX1-9 | KU553439 |
| *atp*B*-rbc*L | *I. sinensis* | TD12-3 | KU553440 |
| *atp*B*-rbc*L | *I. sinensis* | TD13-3 | KU553441 |
| *atp*B*-rbc*L | *I. sinensis* | TD-3 | KU553442 |
| *atp*B*-rbc*L | *I. sinensis* | TD3-1 | KU553443 |
| *atp*B*-rbc*L | *I. sinensis* | TD-4 | KU553444 |
| *atp*B*-rbc*L | *I. sinensis* | TD-5 | KU553445 |
| *atp*B*-rbc*L | *I. sinensis* | TD-6 | KU553446 |
| *atp*B*-rbc*L | *I. sinensis* | TD-7 | KU553447 |
| *atp*B*-rbc*L | *I. sinensis* | TD-8 | KU553448 |
| *atp*B*-rbc*L | *I. sinensis* | TD-9 | KU553449 |
| *atp*B*-rbc*L | *I. sinensis* | TT-1 | KU553450 |
| *atp*B*-rbc*L | *I. sinensis* | TT1-10 | KU553451 |
| *atp*B*-rbc*L | *I. sinensis* | TT1-2 | KU553452 |
| *atp*B*-rbc*L | *I. sinensis* | TT1-4 | KU553453 |
| *atp*B*-rbc*L | *I. sinensis* | TT1-6 | KU553454 |
| *atp*B*-rbc*L | *I. sinensis* | TT-3 | KU553455 |
| *atp*B*-rbc*L | *I. sinensis* | TT-5 | KU553456 |
| *atp*B*-rbc*L | *I. sinensis* | TT-7 | KU553457 |
| *atp*B*-rbc*L | *I. sinensis* | TT-8 | KU553458 |
| *atp*B*-rbc*L | *I. sinensis* | TT-9 | KU553459 |
| *atp*B*-rbc*L | *I. sinensis* | XN-1 | KU553460 |
| *atp*B*-rbc*L | *I. sinensis* | XN-10 | KU553461 |
| *atp*B*-rbc*L | *I. sinensis* | XN1-16 | KU553462 |
| *atp*B*-rbc*L | *I. sinensis* | XN1-2 | KU553463 |
| *atp*B*-rbc*L | *I. sinensis* | XN1-3 | KU553464 |
| *atp*B*-rbc*L | *I. sinensis* | XN1-5 | KU553465 |
| *atp*B*-rbc*L | *I. sinensis* | XN1-8 | KU553466 |
| *atp*B*-rbc*L | *I. sinensis* | XN-4 | KU553467 |
| *atp*B*-rbc*L | *I. sinensis* | XN-7 | KU553468 |
| *atp*B*-rbc*L | *I. sinensis* | XN-9 | KU553469 |
| *trn*S*-trn*G | *I. orientensis* | SY1-1 | KU693470 |
| *trn*S*-trn*G | *I. orientensis* | SY1-10 | KU693471 |
| *trn*S*-trn*G | *I. orientensis* | SY1-22 | KU693472 |
| *trn*S*-trn*G | *I. orientensis* | SY1-3 | KU693473 |
| *trn*S*-trn*G | *I. orientensis* | SY1-4 | KU693474 |
| *trn*S*-trn*G | *I. orientensis* | SY1-5 | KU693475 |
| *trn*S*-trn*G | *I. orientensis* | SY1-6 | KU693476 |
| *trn*S*-trn*G | *I. orientensis* | SY1-7 | KU693477 |
| *trn*S*-trn*G | *I. orientensis* | SY1-8 | KU693478 |
| *trn*S*-trn*G | *I. orientensis* | SY1-9 | KU693479 |
| *trn*S*-trn*G | *I. orientensis* | SY2-10 | KU693480 |
| *trn*S*-trn*G | *I. orientensis* | SY2-3 | KU693481 |
| *trn*S*-trn*G | *I. orientensis* | SY2-38 | KU693482 |
| *trn*S*-trn*G | *I. orientensis* | SY2-4 | KU693483 |
| *trn*S*-trn*G | *I. orientensis* | SY2-42 | KU693484 |
| *trn*S*-trn*G | *I. orientensis* | SY2-5 | KU693485 |
| *trn*S*-trn*G | *I. orientensis* | SY2-6 | KU693486 |
| *trn*S*-trn*G | *I. orientensis* | SY2-7 | KU693487 |
| *trn*S*-trn*G | *I. orientensis* | SY2-8 | KU693488 |
| *trn*S*-trn*G | *I. orientensis* | SY2-9 | KU693489 |
| *trn*S*-trn*G | *I. taiwanensis* | JM1-2 | KU693490 |
| *trn*S*-trn*G | *I. taiwanensis* | JM1-5 | KU693491 |
| *trn*S*-trn*G | *I. taiwanensis* | JM1-7 | KU693492 |
| *trn*S*-trn*G | *I. taiwanensis* | TB-1 | KU693493 |
| *trn*S*-trn*G | *I. taiwanensis* | TB-10 | KU693494 |
| *trn*S*-trn*G | *I. taiwanensis* | TB-3 | KU693495 |
| *trn*S*-trn*G | *I. taiwanensis* | TB-4 | KU693496 |
| *trn*S*-trn*G | *I. taiwanensis* | TB-6 | KU693497 |
| *trn*S*-trn*G | *I. yunguiensis* | NY1 | KU693498 |
| *trn*S*-trn*G | *I. yunguiensis* | NY2 | KU693499 |
| *trn*S*-trn*G | *I. yunguiensis* | PB-10 | KU693500 |
| *trn*S*-trn*G | *I. yunguiensis* | PB-2 | KU693501 |
| *trn*S*-trn*G | *I. yunguiensis* | PB-3 | KU693502 |
| *trn*S*-trn*G | *I. yunguiensis* | PB-6 | KU693503 |
| *trn*S*-trn*G | *I. yunguiensis* | PB-8 | KU693504 |
| *trn*S*-trn*G | *I. yunguiensis* | TC1-3 | KU693505 |
| *trn*S*-trn*G | *I. yunguiensis* | TC1-5 | KU693506 |
| *trn*S*-trn*G | *I. yunguiensis* | TC1-7 | KU693507 |
| *trn*S*-trn*G | *I. yunguiensis* | TC1-8 | KU693508 |
| *trn*S*-trn*G | *I. yunguiensis* | TC1-9 | KU693509 |
| *trn*S*-trn*G | *I. yunguiensis* | TC2-1 | KU693510 |
| *trn*S*-trn*G | *I. yunguiensis* | TC2-5 | KU693511 |
| *trn*S*-trn*G | *I. yunguiensis* | TC2-6 | KU693512 |
| *trn*S*-trn*G | *I. yunguiensis* | TC2-7 | KU693513 |
| *trn*S*-trn*G | *I. yunguiensis* | TC2-8 | KU693514 |
| *trn*S*-trn*G | *I. yunguiensis* | HF1 | KU693515 |
| *trn*S*-trn*G | *I. yunguiensis* | HF3 | KU693516 |
| *trn*S*-trn*G | *I. yunguiensis* | HF4 | KU693517 |
| *trn*S*-trn*G | *I. yunguiensis* | HF5 | KU693518 |
| *trn*S*-trn*G | *I. sinensis* | HT10 | KU693519 |
| *trn*S*-trn*G | *I. sinensis* | HT1-1 | KU693520 |
| *trn*S*-trn*G | *I. sinensis* | HT1-2 | KU693521 |
| *trn*S*-trn*G | *I. sinensis* | HT1-3 | KU693522 |
| *trn*S*-trn*G | *I. sinensis* | HT1-4 | KU693523 |
| *trn*S*-trn*G | *I. sinensis* | HT1-5 | KU693524 |
| *trn*S*-trn*G | *I. sinensis* | HT1-6 | KU693525 |
| *trn*S*-trn*G | *I. sinensis* | HT1-7 | KU693526 |
| *trn*S*-trn*G | *I. sinensis* | HT1-8 | KU693527 |
| *trn*S*-trn*G | *I. sinensis* | HT1-9 | KU693528 |
| *trn*S*-trn*G | *I. sinensis* | JD1-1 | KU693529 |
| *trn*S*-trn*G | *I. sinensis* | JD1-10 | KU693530 |
| *trn*S*-trn*G | *I. sinensis* | JD1-2 | KU693531 |
| *trn*S*-trn*G | *I. sinensis* | JD1-3 | KU693532 |
| *trn*S*-trn*G | *I. sinensis* | JD1-4 | KU693533 |
| *trn*S*-trn*G | *I. sinensis* | JD1-5 | KU693534 |
| *trn*S*-trn*G | *I. sinensis* | JD1-6 | KU693535 |
| *trn*S*-trn*G | *I. sinensis* | JD1-7 | KU693536 |
| *trn*S*-trn*G | *I. sinensis* | JD1-8 | KU693537 |
| *trn*S*-trn*G | *I. sinensis* | JD1-9 | KU693538 |
| *trn*S*-trn*G | *I. sinensis* | JD2-1 | KU693539 |
| *trn*S*-trn*G | *I. sinensis* | JD2-3 | KU693540 |
| *trn*S*-trn*G | *I. sinensis* | JD2-4 | KU693541 |
| *trn*S*-trn*G | *I. sinensis* | JD2-42 | KU693542 |
| *trn*S*-trn*G | *I. sinensis* | JD2-49 | KU693543 |
| *trn*S*-trn*G | *I. sinensis* | JD2-5 | KU693544 |
| *trn*S*-trn*G | *I. sinensis* | JD2-6 | KU693545 |
| *trn*S*-trn*G | *I. sinensis* | JD2-7 | KU693546 |
| *trn*S*-trn*G | *I. sinensis* | JD2-8 | KU693547 |
| *trn*S*-trn*G | *I. sinensis* | JD2-9 | KU693548 |
| *trn*S*-trn*G | *I. sinensis* | NX1-1 | KU693549 |
| *trn*S*-trn*G | *I. sinensis* | NX1-10 | KU693550 |
| *trn*S*-trn*G | *I. sinensis* | NX1-2 | KU693551 |
| *trn*S*-trn*G | *I. sinensis* | NX1-3 | KU693552 |
| *trn*S*-trn*G | *I. sinensis* | NX1-4 | KU693553 |
| *trn*S*-trn*G | *I. sinensis* | NX1-5 | KU693554 |
| *trn*S*-trn*G | *I. sinensis* | NX1-6 | KU693555 |
| *trn*S*-trn*G | *I. sinensis* | NX1-7 | KU693556 |
| *trn*S*-trn*G | *I. sinensis* | NX1-8 | KU693557 |
| *trn*S*-trn*G | *I. sinensis* | NX1-9 | KU693558 |
| *trn*S*-trn*G | *I. sinensis* | TD12-3 | KU693559 |
| *trn*S*-trn*G | *I. sinensis* | TD13-3 | KU693560 |
| *trn*S*-trn*G | *I. sinensis* | TD-3 | KU693561 |
| *trn*S*-trn*G | *I. sinensis* | TD3-1 | KU693562 |
| *trn*S*-trn*G | *I. sinensis* | TD-4 | KU693563 |
| *trn*S*-trn*G | *I. sinensis* | TD-5 | KU693564 |
| *trn*S*-trn*G | *I. sinensis* | TD-6 | KU693565 |
| *trn*S*-trn*G | *I. sinensis* | TD-7 | KU693566 |
| *trn*S*-trn*G | *I. sinensis* | TD-8 | KU693567 |
| *trn*S*-trn*G | *I. sinensis* | TD-9 | KU693568 |
| *trn*S*-trn*G | *I. sinensis* | TT-1 | KU693569 |
| *trn*S*-trn*G | *I. sinensis* | TT1-10 | KU693570 |
| *trn*S*-trn*G | *I. sinensis* | TT1-2 | KU693571 |
| *trn*S*-trn*G | *I. sinensis* | TT1-4 | KU693572 |
| *trn*S*-trn*G | *I. sinensis* | TT1-6 | KU693573 |
| *trn*S*-trn*G | *I. sinensis* | TT-3 | KU693574 |
| *trn*S*-trn*G | *I. sinensis* | TT-5 | KU693575 |
| *trn*S*-trn*G | *I. sinensis* | TT-7 | KU693576 |
| *trn*S*-trn*G | *I. sinensis* | TT-8 | KU693577 |
| *trn*S*-trn*G | *I. sinensis* | TT-9 | KU693578 |
| *trn*S*-trn*G | *I. sinensis* | XN-1 | KU693579 |
| *trn*S*-trn*G | *I. sinensis* | XN-10 | KU693580 |
| *trn*S*-trn*G | *I. sinensis* | XN1-16 | KU693581 |
| *trn*S*-trn*G | *I. sinensis* | XN1-2 | KU693582 |
| *trn*S*-trn*G | *I. sinensis* | XN1-3 | KU693583 |
| *trn*S*-trn*G | *I. sinensis* | XN1-5 | KU693584 |
| *trn*S*-trn*G | *I. sinensis* | XN1-8 | KU693585 |
| *trn*S*-trn*G | *I. sinensis* | XN-4 | KU693586 |
| *trn*S*-trn*G | *I. sinensis* | XN-7 | KU693587 |
| *trn*S*-trn*G | *I. sinensis* | XN-9 | KU693588 |
| *pet*L*-psb*E | *I. taiwanensis* | TB-1 | KU600955 |
| *pet*L*-psb*E | *I. taiwanensis* | TB-10 | KU600956 |
| *pet*L*-psb*E | *I. taiwanensis* | TB-3 | KU600957 |
| *pet*L*-psb*E | *I. taiwanensis* | TB-4 | KU600958 |
| *pet*L*-psb*E | *I. taiwanensis* | TB-6 | KU600959 |
| *pet*L*-psb*E | *I. taiwanensis* | JM1-2 | KU600960 |
| *pet*L*-psb*E | *I. taiwanensis* | JM1-5 | KU600961 |
| *pet*L*-psb*E | *I. taiwanensis* | JM1-7 | KU600962 |
| *pet*L*-psb*E | *I. yunguiensis* | HF1 | KU600963 |
| *pet*L*-psb*E | *I. yunguiensis* | HF3 | KU600964 |
| *pet*L*-psb*E | *I. yunguiensis* | HF4 | KU600965 |
| *pet*L*-psb*E | *I. yunguiensis* | HF5 | KU600966 |
| *pet*L*-psb*E | *I. yunguiensis* | NY1 | KU600967 |
| *pet*L*-psb*E | *I. yunguiensis* | NY2 | KU600968 |
| *pet*L*-psb*E | *I. yunguiensis* | PB-10 | KU600969 |
| *pet*L*-psb*E | *I. yunguiensis* | PB-2 | KU600970 |
| *pet*L*-psb*E | *I. yunguiensis* | PB-3 | KU600971 |
| *pet*L*-psb*E | *I. yunguiensis* | PB-6 | KU600972 |
| *pet*L*-psb*E | *I. yunguiensis* | PB-8 | KU600973 |
| *pet*L*-psb*E | *I. yunguiensis* | TC1-3 | KU600974 |
| *pet*L*-psb*E | *I. yunguiensis* | TC1-5 | KU600975 |
| *pet*L*-psb*E | *I. yunguiensis* | TC1-7 | KU600976 |
| *pet*L*-psb*E | *I. yunguiensis* | TC1-8 | KU600977 |
| *pet*L*-psb*E | *I. yunguiensis* | TC1-9 | KU600978 |
| *pet*L*-psb*E | *I. yunguiensis* | TC2-1 | KU600979 |
| *pet*L*-psb*E | *I. yunguiensis* | TC2-5 | KU600980 |
| *pet*L*-psb*E | *I. yunguiensis* | TC2-6 | KU600981 |
| *pet*L*-psb*E | *I. yunguiensis* | TC2-7 | KU600982 |
| *pet*L*-psb*E | *I. yunguiensis* | TC2-8 | KU600983 |
| *pet*L*-psb*E | *I. orientensis* | SY1-1 | KU600985 |
| *pet*L*-psb*E | *I. orientensis* | SY1-10 | KU600986 |
| *pet*L*-psb*E | *I. orientensis* | SY1-22 | KU600987 |
| *pet*L*-psb*E | *I. orientensis* | SY1-3 | KU600988 |
| *pet*L*-psb*E | *I. orientensis* | SY1-4 | KU600989 |
| *pet*L*-psb*E | *I. orientensis* | SY1-5 | KU600990 |
| *pet*L*-psb*E | *I. orientensis* | SY1-6 | KU600991 |
| *pet*L*-psb*E | *I. orientensis* | SY1-7 | KU600992 |
| *pet*L*-psb*E | *I. orientensis* | SY1-8 | KU600993 |
| *pet*L*-psb*E | *I. orientensis* | SY1-9 | KU600994 |
| *pet*L*-psb*E | *I. orientensis* | SY2-10 | KU600995 |
| *pet*L*-psb*E | *I. orientensis* | SY2-3 | KU600996 |
| *pet*L*-psb*E | *I. orientensis* | SY2-38 | KU600997 |
| *pet*L*-psb*E | *I. orientensis* | SY2-4 | KU600998 |
| *pet*L*-psb*E | *I. orientensis* | SY2-42 | KU600999 |
| *pet*L*-psb*E | *I. orientensis* | SY2-5 | KU601000 |
| *pet*L*-psb*E | *I. orientensis* | SY2-6 | KU601001 |
| *pet*L*-psb*E | *I. orientensis* | SY2-7 | KU601002 |
| *pet*L*-psb*E | *I. orientensis* | SY2-8 | KU601003 |
| *pet*L*-psb*E | *I. orientensis* | SY2-9 | KU601004 |
| *pet*L*-psb*E | *I. sinensis* | HT10 | KU601005 |
| *pet*L*-psb*E | *I. sinensis* | HT1-1 | KU601006 |
| *pet*L*-psb*E | *I. sinensis* | HT1-2 | KU601007 |
| *pet*L*-psb*E | *I. sinensis* | HT1-3 | KU601008 |
| *pet*L*-psb*E | *I. sinensis* | HT1-4 | KU601009 |
| *pet*L*-psb*E | *I. sinensis* | HT1-5 | KU601010 |
| *pet*L*-psb*E | *I. sinensis* | HT1-6 | KU601011 |
| *pet*L*-psb*E | *I. sinensis* | HT1-7 | KU601012 |
| *pet*L*-psb*E | *I. sinensis* | HT1-8 | KU601013 |
| *pet*L*-psb*E | *I. sinensis* | HT1-9 | KU601014 |
| *pet*L*-psb*E | *I. sinensis* | JD1-1 | KU601015 |
| *pet*L*-psb*E | *I. sinensis* | JD1-10 | KU601016 |
| *pet*L*-psb*E | *I. sinensis* | JD1-2 | KU601017 |
| *pet*L*-psb*E | *I. sinensis* | JD1-3 | KU601018 |
| *pet*L*-psb*E | *I. sinensis* | JD1-4 | KU601019 |
| *pet*L*-psb*E | *I. sinensis* | JD1-5 | KU601020 |
| *pet*L*-psb*E | *I. sinensis* | JD1-6 | KU601021 |
| *pet*L*-psb*E | *I. sinensis* | JD1-7 | KU601022 |
| *pet*L*-psb*E | *I. sinensis* | JD1-8 | KU601023 |
| *pet*L*-psb*E | *I. sinensis* | JD1-9 | KU601024 |
| *pet*L*-psb*E | *I. sinensis* | JD2-1 | KU601025 |
| *pet*L*-psb*E | *I. sinensis* | JD2-3 | KU601026 |
| *pet*L*-psb*E | *I. sinensis* | JD2-4 | KU601027 |
| *pet*L*-psb*E | *I. sinensis* | JD2-42 | KU601028 |
| *pet*L*-psb*E | *I. sinensis* | JD2-49 | KU601029 |
| *pet*L*-psb*E | *I. sinensis* | JD2-5 | KU601030 |
| *pet*L*-psb*E | *I. sinensis* | JD2-6 | KU601031 |
| *pet*L*-psb*E | *I. sinensis* | JD2-7 | KU601032 |
| *pet*L*-psb*E | *I. sinensis* | JD2-8 | KU601033 |
| *pet*L*-psb*E | *I. sinensis* | JD2-9 | KU601034 |
| *pet*L*-psb*E | *I. sinensis* | NX1-1 | KU601035 |
| *pet*L*-psb*E | *I. sinensis* | NX1-10 | KU601036 |
| *pet*L*-psb*E | *I. sinensis* | NX1-2 | KU601037 |
| *pet*L*-psb*E | *I. sinensis* | NX1-3 | KU601038 |
| *pet*L*-psb*E | *I. sinensis* | NX1-4 | KU601039 |
| *pet*L*-psb*E | *I. sinensis* | NX1-5 | KU601040 |
| *pet*L*-psb*E | *I. sinensis* | NX1-6 | KU601041 |
| *pet*L*-psb*E | *I. sinensis* | NX1-7 | KU601042 |
| *pet*L*-psb*E | *I. sinensis* | NX1-8 | KU601043 |
| *pet*L*-psb*E | *I. sinensis* | NX1-9 | KU601044 |
| *pet*L*-psb*E | *I. sinensis* | TD12-3 | KU601045 |
| *pet*L*-psb*E | *I. sinensis* | TD13-3 | KU601046 |
| *pet*L*-psb*E | *I. sinensis* | TD-3 | KU601047 |
| *pet*L*-psb*E | *I. sinensis* | TD3-1 | KU601048 |
| *pet*L*-psb*E | *I. sinensis* | TD-4 | KU601049 |
| *pet*L*-psb*E | *I. sinensis* | TD-5 | KU601050 |
| *pet*L*-psb*E | *I. sinensis* | TD-6 | KU601051 |
| *pet*L*-psb*E | *I. sinensis* | TD-7 | KU601052 |
| *pet*L*-psb*E | *I. sinensis* | TD-8 | KU601053 |
| *pet*L*-psb*E | *I. sinensis* | TD-9 | KU601054 |
| *pet*L*-psb*E | *I. sinensis* | TT-1 | KU601055 |
| *pet*L*-psb*E | *I. sinensis* | TT1-10 | KU601056 |
| *pet*L*-psb*E | *I. sinensis* | TT1-2 | KU601057 |
| *pet*L*-psb*E | *I. sinensis* | TT1-4 | KU601058 |
| *pet*L*-psb*E | *I. sinensis* | TT1-6 | KU601059 |
| *pet*L*-psb*E | *I. sinensis* | TT-3 | KU601060 |
| *pet*L*-psb*E | *I. sinensis* | TT-5 | KU601061 |
| *pet*L*-psb*E | *I. sinensis* | TT-7 | KU601062 |
| *pet*L*-psb*E | *I. sinensis* | TT-8 | KU601063 |
| *pet*L*-psb*E | *I. sinensis* | TT-9 | KU601064 |
| *pet*L*-psb*E | *I. sinensis* | XN-1 | KU601065 |
| *pet*L*-psb*E | *I. sinensis* | XN-10 | KU601066 |
| *pet*L*-psb*E | *I. sinensis* | XN1-16 | KU601067 |
| *pet*L*-psb*E | *I. sinensis* | XN1-2 | KU601068 |
| *pet*L*-psb*E | *I. sinensis* | XN1-3 | KU601069 |
| *pet*L*-psb*E | *I. sinensis* | XN1-5 | KU601070 |
| *pet*L*-psb*E | *I. sinensis* | XN1-8 | KU601071 |
| *pet*L*-psb*E | *I. sinensis* | XN-4 | KU601072 |
| *pet*L*-psb*E | *I. sinensis* | XN-7 | KU601073 |
| *pet*L*-psb*E | *I. sinensis* | XN-9 | KU601074 |
| *ycf66* | *I. sinensis* | HT10 | KU601075 |
| *ycf66* | *I. sinensis* | HT1-1 | KU601076 |
| *ycf66* | *I. sinensis* | HT1-2 | KU601077 |
| *ycf66* | *I. sinensis* | HT1-3 | KU601078 |
| *ycf66* | *I. sinensis* | HT1-4 | KU601079 |
| *ycf66* | *I. sinensis* | HT1-5 | KU601080 |
| *ycf66* | *I. sinensis* | HT1-6 | KU601081 |
| *ycf66* | *I. sinensis* | HT1-7 | KU601082 |
| *ycf66* | *I. sinensis* | HT1-8 | KU601083 |
| *ycf66* | *I. sinensis* | HT1-9 | KU601084 |
| *ycf66* | *I. sinensis* | JD1-1 | KU601085 |
| *ycf66* | *I. sinensis* | JD1-10 | KU601086 |
| *ycf66* | *I. sinensis* | JD1-2 | KU601087 |
| *ycf66* | *I. sinensis* | JD1-3 | KU601088 |
| *ycf66* | *I. sinensis* | JD1-4 | KU601089 |
| *ycf66* | *I. sinensis* | JD1-5 | KU601090 |
| *ycf66* | *I. sinensis* | JD1-6 | KU601091 |
| *ycf66* | *I. sinensis* | JD1-7 | KU601092 |
| *ycf66* | *I. sinensis* | JD1-8 | KU601093 |
| *ycf66* | *I. sinensis* | JD1-9 | KU601094 |
| *ycf66* | *I. sinensis* | JD2-1 | KU601095 |
| *ycf66* | *I. sinensis* | JD2-3 | KU601096 |
| *ycf66* | *I. sinensis* | JD2-4 | KU601097 |
| *ycf66* | *I. sinensis* | JD2-42 | KU601098 |
| *ycf66* | *I. sinensis* | JD2-49 | KU601099 |
| *ycf66* | *I. sinensis* | JD2-5 | KU601100 |
| *ycf66* | *I. sinensis* | JD2-6 | KU601101 |
| *ycf66* | *I. sinensis* | JD2-7 | KU601102 |
| *ycf66* | *I. sinensis* | JD2-8 | KU601103 |
| *ycf66* | *I. sinensis* | JD2-9 | KU601104 |
| *ycf66* | *I. sinensis* | NX1-1 | KU601105 |
| *ycf66* | *I. sinensis* | NX1-10 | KU601106 |
| *ycf66* | *I. sinensis* | NX1-2 | KU601107 |
| *ycf66* | *I. sinensis* | NX1-3 | KU601108 |
| *ycf66* | *I. sinensis* | NX1-4 | KU601109 |
| *ycf66* | *I. sinensis* | NX1-5 | KU601110 |
| *ycf66* | *I. sinensis* | NX1-6 | KU601111 |
| *ycf66* | *I. sinensis* | NX1-7 | KU601112 |
| *ycf66* | *I. sinensis* | NX1-8 | KU601113 |
| *ycf66* | *I. sinensis* | NX1-9 | KU601114 |
| *ycf66* | *I. sinensis* | TD12-3 | KU601115 |
| *ycf66* | *I. sinensis* | TD13-3 | KU601116 |
| *ycf66* | *I. sinensis* | TD-3 | KU601117 |
| *ycf66* | *I. sinensis* | TD3-1 | KU601118 |
| *ycf66* | *I. sinensis* | TD-4 | KU601119 |
| *ycf66* | *I. sinensis* | TD-5 | KU601120 |
| *ycf66* | *I. sinensis* | TD-6 | KU601121 |
| *ycf66* | *I. sinensis* | TD-7 | KU601122 |
| *ycf66* | *I. sinensis* | TD-8 | KU601123 |
| *ycf66* | *I. sinensis* | TD-9 | KU601124 |
| *ycf66* | *I. sinensis* | TT-1 | KU601125 |
| *ycf66* | *I. sinensis* | TT1-10 | KU601126 |
| *ycf66* | *I. sinensis* | TT1-2 | KU601127 |
| *ycf66* | *I. sinensis* | TT1-4 | KU601128 |
| *ycf66* | *I. sinensis* | TT1-6 | KU601129 |
| *ycf66* | *I. sinensis* | TT-3 | KU601130 |
| *ycf66* | *I. sinensis* | TT-5 | KU601131 |
| *ycf66* | *I. sinensis* | TT-7 | KU601132 |
| *ycf66* | *I. sinensis* | TT-8 | KU601133 |
| *ycf66* | *I. sinensis* | TT-9 | KU601134 |
| *ycf66* | *I. sinensis* | XN-1 | KU601135 |
| *ycf66* | *I. sinensis* | XN-10 | KU601136 |
| *ycf66* | *I. sinensis* | XN1-16 | KU601137 |
| *ycf66* | *I. sinensis* | XN1-2 | KU601138 |
| *ycf66* | *I. sinensis* | XN1-3 | KU601139 |
| *ycf66* | *I. sinensis* | XN1-5 | KU601140 |
| *ycf66* | *I. sinensis* | XN1-8 | KU601141 |
| *ycf66* | *I. sinensis* | XN-4 | KU601142 |
| *ycf66* | *I. sinensis* | XN-7 | KU601143 |
| *ycf66* | *I. sinensis* | XN-9 | KU601144 |
| *ycf66* | *I. taiwanensis* | TB-1 | KU601145 |
| *ycf66* | *I. taiwanensis* | TB-10 | KU601146 |
| *ycf66* | *I. taiwanensis* | TB-3 | KU601147 |
| *ycf66* | *I. taiwanensis* | TB-4 | KU601148 |
| *ycf66* | *I. taiwanensis* | TB-6 | KU601149 |
| *ycf66* | *I. taiwanensis* | JM1-2 | KU601150 |
| *ycf66* | *I. taiwanensis* | JM1-5 | KU601151 |
| *ycf66* | *I. taiwanensis* | JM1-7 | KU601152 |
| *ycf66* | *I. yunguiensis* | HF1 | KU601153 |
| *ycf66* | *I. yunguiensis* | HF3 | KU601154 |
| *ycf66* | *I. yunguiensis* | HF4 | KU601155 |
| *ycf66* | *I. yunguiensis* | HF5 | KU601156 |
| *ycf66* | *I. yunguiensis* | NY1 | KU601157 |
| *ycf66* | *I. yunguiensis* | NY2 | KU601158 |
| *ycf66* | *I. yunguiensis* | PB-10 | KU601159 |
| *ycf66* | *I. yunguiensis* | PB-2 | KU601160 |
| *ycf66* | *I. yunguiensis* | PB-3 | KU601161 |
| *ycf66* | *I. yunguiensis* | PB-6 | KU601162 |
| *ycf66* | *I. yunguiensis* | PB-8 | KU601163 |
| *ycf66* | *I. yunguiensis* | TC1-3 | KU601164 |
| *ycf66* | *I. yunguiensis* | TC1-5 | KU601165 |
| *ycf66* | *I. yunguiensis* | TC1-7 | KU601166 |
| *ycf66* | *I. yunguiensis* | TC1-8 | KU601167 |
| *ycf66* | *I. yunguiensis* | TC1-9 | KU601168 |
| *ycf66* | *I. yunguiensis* | TC2-1 | KU601169 |
| *ycf66* | *I. yunguiensis* | TC2-5 | KU601170 |
| *ycf66* | *I. yunguiensis* | TC2-6 | KU601171 |
| *ycf66* | *I. yunguiensis* | TC2-7 | KU601172 |
| *ycf66* | *I. yunguiensis* | TC2-8 | KU601173 |
| *ycf66* | *I. orientensis* | SY1-1 | KU601175 |
| *ycf66* | *I. orientensis* | SY1-10 | KU601176 |
| *ycf66* | *I. orientensis* | SY1-22 | KU601177 |
| *ycf66* | *I. orientensis* | SY1-3 | KU601178 |
| *ycf66* | *I. orientensis* | SY1-4 | KU601179 |
| *ycf66* | *I. orientensis* | SY1-5 | KU601180 |
| *ycf66* | *I. orientensis* | SY1-6 | KU601181 |
| *ycf66* | *I. orientensis* | SY1-7 | KU601182 |
| *ycf66* | *I. orientensis* | SY1-8 | KU601183 |
| *ycf66* | *I. orientensis* | SY1-9 | KU601184 |
| *ycf66* | *I. orientensis* | SY2-10 | KU601185 |
| *ycf66* | *I. orientensis* | SY2-3 | KU601186 |
| *ycf66* | *I. orientensis* | SY2-38 | KU601187 |
| *ycf66* | *I. orientensis* | SY2-4 | KU601188 |
| *ycf66* | *I. orientensis* | SY2-42 | KU601189 |
| *ycf66* | *I. orientensis* | SY2-5 | KU601190 |
| *ycf66* | *I. orientensis* | SY2-6 | KU601191 |
| *ycf66* | *I. orientensis* | SY2-7 | KU601192 |
| *ycf66* | *I. orientensis* | SY2-8 | KU601193 |
| *ycf66* | *I. orientensis* | SY2-9 | KU601194 |
