# Supplementary material for: The speciation and adaptation of the polyploids: a case study of the Chinese Isoetes L. diploid-polyploid complex
Source: BMC Evol Biol. 2020 Sep 14;20:118. doi: 10.1186/s12862-020-01687-4 (PMC7490897; doi:10.1186/s12862-020-01687-4)
Supplement: Supplementary file 1 — Additional file 1: Table S1. The serial numbers of plastid DNA sequences in this study. Table S2. The serial numbers of nuclear DNA sequences in this study. Table S3. Haplotypes information of nuclear DNA data. Table S4. Haplotypes information of cpDNA data. Table S5. Location records used for ecological niche modeling. Table S6. Results of the nonparametric Kruskal test applied for the populations whose maternal contributor are different in the allopolyploid populations of I.sinensis. [file 12862_2020_1687_MOESM1_ESM.zip › Table S2.docx]

Table. S2. The serial numbers of nuclear DNA sequences in this study, including sequences of Chinese *Isoëtes* complex and out group *I. hypsophila*.

| Gene name | Species | Sequence name | Serial name |
| --- | --- | --- | --- |
| *LFY* | *I. taiwanensis* | TB-1 | KU736964 |
| *LFY* | *I. taiwanensis* | TB1-102 | KU736965 |
| *LFY* | *I. taiwanensis* | TB1-104 | KU736966 |
| *LFY* | *I. taiwanensis* | TB1-105 | KU736967 |
| *LFY* | *I. taiwanensis* | TB1-108 | KU736968 |
| *LFY* | *I. taiwanensis* | TB1-109 | KU736969 |
| *LFY* | *I. taiwanensis* | TB111 | KU736970 |
| *LFY* | *I. taiwanensis* | TB1-11 | KU736971 |
| *LFY* | *I. taiwanensis* | TB1-12 | KU736972 |
| *LFY* | *I. taiwanensis* | TB1-13 | KU736973 |
| *LFY* | *I. taiwanensis* | TB1-31 | KU736974 |
| *LFY* | *I. taiwanensis* | TB1-33 | KU736975 |
| *LFY* | *I. taiwanensis* | TB1-34 | KU736976 |
| *LFY* | *I. taiwanensis* | TB1-35 | KU736977 |
| *LFY* | *I. taiwanensis* | TB1-410 | KU736978 |
| *LFY* | *I. taiwanensis* | TB1-413 | KU736979 |
| *LFY* | *I. taiwanensis* | TB1-42 | KU736980 |
| *LFY* | *I. taiwanensis* | TB1-44 | KU736981 |
| *LFY* | *I. taiwanensis* | TB1-49 | KU736982 |
| *LFY* | *I. taiwanensis* | TB1-63 | KU736983 |
| *LFY* | *I. taiwanensis* | TB1-64 | KU736984 |
| *LFY* | *I. taiwanensis* | TB1-65 | KU736985 |
| *LFY* | *I. taiwanensis* | TB1-66 | KU736986 |
| *LFY* | *I. taiwanensis* | TB311 | KU736987 |
| *LFY* | *I. taiwanensis* | TB67 | KU736988 |
| *LFY* | *I. taiwanensis* | JM106 | KU736989 |
| *LFY* | *I. taiwanensis* | JM11 | KU736990 |
| *LFY* | *I. taiwanensis* | JM1-128 | KU736991 |
| *LFY* | *I. taiwanensis* | JM12 | KU736992 |
| *LFY* | *I. taiwanensis* | JM1-213 | KU736993 |
| *LFY* | *I. taiwanensis* | JM1-214 | KU736994 |
| *LFY* | *I. taiwanensis* | JM1-25 | KU736995 |
| *LFY* | *I. taiwanensis* | JM1-27 | KU736996 |
| *LFY* | *I. taiwanensis* | JM13 | KU736997 |
| *LFY* | *I. taiwanensis* | JM1-31 | KU736998 |
| *LFY* | *I. taiwanensis* | JM1-318 | KU736999 |
| *LFY* | *I. taiwanensis* | JM1-319 | KU737000 |
| *LFY* | *I. taiwanensis* | JM1-32 | KU737001 |
| *LFY* | *I. taiwanensis* | JM1-34 | KU737002 |
| *LFY* | *I. taiwanensis* | JM1-51 | KU737003 |
| *LFY* | *I. taiwanensis* | JM1-53 | KU737004 |
| *LFY* | *I. taiwanensis* | JM1-54 | KU737005 |
| *LFY* | *I. taiwanensis* | JM1-55 | KU737006 |
| *LFY* | *I. taiwanensis* | JM1-56 | KU737007 |
| *LFY* | *I. taiwanensis* | JM1-71 | KU737008 |
| *LFY* | *I. taiwanensis* | JM1-710 | KU737009 |
| *LFY* | *I. taiwanensis* | JM1-718 | KU737010 |
| *LFY* | *I. taiwanensis* | JM1-73 | KU737011 |
| *LFY* | *I. taiwanensis* | JM1-75 | KU737012 |
| *LFY* | *I. taiwanensis* | JM218 | KU737013 |
| *LFY* | *I. yunguiensis* | HF11 | KU737014 |
| *LFY* | *I. yunguiensis* | HF12 | KU737015 |
| *LFY* | *I. yunguiensis* | HF13 | KU737016 |
| *LFY* | *I. yunguiensis* | HF1-36 | KU737017 |
| *LFY* | *I. yunguiensis* | HF14 | KU737018 |
| *LFY* | *I. yunguiensis* | HF15 | KU737019 |
| *LFY* | *I. yunguiensis* | HF1-56 | KU737020 |
| *LFY* | *I. yunguiensis* | HF31 | KU737021 |
| *LFY* | *I. yunguiensis* | HF310 | KU737022 |
| *LFY* | *I. yunguiensis* | HF311 | KU737023 |
| *LFY* | *I. yunguiensis* | HF35 | KU737024 |
| *LFY* | *I. yunguiensis* | HF414 | KU737025 |
| *LFY* | *I. yunguiensis* | HF417 | KU737026 |
| *LFY* | *I. yunguiensis* | HF43 | KU737027 |
| *LFY* | *I. yunguiensis* | HF44 | KU737028 |
| *LFY* | *I. yunguiensis* | HF45 | KU737029 |
| *LFY* | *I. yunguiensis* | HF51 | KU737030 |
| *LFY* | *I. yunguiensis* | HF52 | KU737031 |
| *LFY* | *I. yunguiensis* | HF57 | KU737032 |
| *LFY* | *I. yunguiensis* | HF59 | KU737033 |
| *LFY* | *I. yunguiensis* | NY11 | KU737034 |
| *LFY* | *I. yunguiensis* | NY13 | KU737035 |
| *LFY* | *I. yunguiensis* | NY14 | KU737036 |
| *LFY* | *I. yunguiensis* | NY15 | KU737037 |
| *LFY* | *I. yunguiensis* | NY17 | KU737038 |
| *LFY* | *I. yunguiensis* | NY21 | KU737039 |
| *LFY* | *I. yunguiensis* | NY22 | KU737040 |
| *LFY* | *I. yunguiensis* | NY24 | KU737041 |
| *LFY* | *I. yunguiensis* | NY25 | KU737042 |
| *LFY* | *I. yunguiensis* | NY27 | KU737043 |
| *LFY* | *I. yunguiensis* | PB10-1 | KU737044 |
| *LFY* | *I. yunguiensis* | PB1017 | KU737045 |
| *LFY* | *I. yunguiensis* | PB1-101 | KU737046 |
| *LFY* | *I. yunguiensis* | PB1-1010 | KU737047 |
| *LFY* | *I. yunguiensis* | PB1-1015 | KU737048 |
| *LFY* | *I. yunguiensis* | PB1-21 | KU737049 |
| *LFY* | *I. yunguiensis* | PB1-25 | KU737050 |
| *LFY* | *I. yunguiensis* | PB1-26 | KU737051 |
| *LFY* | *I. yunguiensis* | PB1-31 | KU737052 |
| *LFY* | *I. yunguiensis* | PB1-32 | KU737053 |
| *LFY* | *I. yunguiensis* | PB1-36 | KU737054 |
| *LFY* | *I. yunguiensis* | PB1-37 | KU737055 |
| *LFY* | *I. yunguiensis* | PB1-38 | KU737056 |
| *LFY* | *I. yunguiensis* | PB1-613 | KU737057 |
| *LFY* | *I. yunguiensis* | PB1-615 | KU737058 |
| *LFY* | *I. yunguiensis* | PB1-62 | KU737059 |
| *LFY* | *I. yunguiensis* | PB1-63 | KU737060 |
| *LFY* | *I. yunguiensis* | PB6-17 | KU737061 |
| *LFY* | *I. yunguiensis* | PB1-811 | KU737062 |
| *LFY* | *I. yunguiensis* | PB1-812 | KU737063 |
| *LFY* | *I. yunguiensis* | PB1-813 | KU737064 |
| *LFY* | *I. yunguiensis* | PB1-85 | KU737065 |
| *LFY* | *I. yunguiensis* | PB1-87 | KU737066 |
| *LFY* | *I. yunguiensis* | PB218 | KU737067 |
| *LFY* | *I. yunguiensis* | PB220 | KU737068 |
| *LFY* | *I. yunguiensis* | TTC1-33 | KU737069 |
| *LFY* | *I. yunguiensis* | TTC1-34 | KU737070 |
| *LFY* | *I. yunguiensis* | TTC1-51 | KU737071 |
| *LFY* | *I. yunguiensis* | TTC1-52 | KU737072 |
| *LFY* | *I. yunguiensis* | TTC1-72 | KU737073 |
| *LFY* | *I. yunguiensis* | TTC1-81 | KU737074 |
| *LFY* | *I. yunguiensis* | TTC1-82 | KU737075 |
| *LFY* | *I. yunguiensis* | tc1-3 | KU737076 |
| *LFY* | *I. yunguiensis* | TC1-3C | KU737077 |
| *LFY* | *I. yunguiensis* | TC1-3F | KU737078 |
| *LFY* | *I. yunguiensis* | tc1-5 | KU737079 |
| *LFY* | *I. yunguiensis* | TC1-5B | KU737080 |
| *LFY* | *I. yunguiensis* | TC1-5C | KU737081 |
| *LFY* | *I. yunguiensis* | tc1-7 | KU737082 |
| *LFY* | *I. yunguiensis* | TC1-74 | KU737083 |
| *LFY* | *I. yunguiensis* | TC1-7B | KU737084 |
| *LFY* | *I. yunguiensis* | TC1-7C | KU737085 |
| *LFY* | *I. yunguiensis* | tc1-8 | KU737086 |
| *LFY* | *I. yunguiensis* | TC1-8B | KU737087 |
| *LFY* | *I. yunguiensis* | TC1-8D | KU737088 |
| *LFY* | *I. yunguiensis* | tc1-9 | KU737089 |
| *LFY* | *I. yunguiensis* | TC1-98 | KU737090 |
| *LFY* | *I. yunguiensis* | TC1-99 | KU737091 |
| *LFY* | *I. yunguiensis* | TC1-9B | KU737092 |
| *LFY* | *I. yunguiensis* | TC1-9C | KU737093 |
| *LFY* | *I. yunguiensis* | TC2-12 | KU737094 |
| *LFY* | *I. yunguiensis* | TC2-14 | KU737095 |
| *LFY* | *I. yunguiensis* | TC2-15 | KU737096 |
| *LFY* | *I. yunguiensis* | TC2-17 | KU737097 |
| *LFY* | *I. yunguiensis* | TC2-18 | KU737098 |
| *LFY* | *I. yunguiensis* | TC2-53 | KU737099 |
| *LFY* | *I. yunguiensis* | TC2-54 | KU737100 |
| *LFY* | *I. yunguiensis* | TC2-55 | KU737101 |
| *LFY* | *I. yunguiensis* | TC2-56 | KU737102 |
| *LFY* | *I. yunguiensis* | TC2-57 | KU737103 |
| *LFY* | *I. yunguiensis* | TC2-61 | KU737104 |
| *LFY* | *I. yunguiensis* | TC2-62 | KU737105 |
| *LFY* | *I. yunguiensis* | TC2-63 | KU737106 |
| *LFY* | *I. yunguiensis* | TC2-68 | KU737107 |
| *LFY* | *I. yunguiensis* | TC2-69 | KU737108 |
| *LFY* | *I. yunguiensis* | TC2-71 | KU737109 |
| *LFY* | *I. yunguiensis* | TC2-72 | KU737110 |
| *LFY* | *I. yunguiensis* | TC2-73 | KU737111 |
| *LFY* | *I. yunguiensis* | TC2-74 | KU737112 |
| *LFY* | *I. yunguiensis* | TC2-76 | KU737113 |
| *LFY* | *I. yunguiensis* | TC2-81 | KU737114 |
| *LFY* | *I. yunguiensis* | TC2-82 | KU737115 |
| *LFY* | *I. yunguiensis* | TC2-83 | KU737116 |
| *LFY* | *I. yunguiensis* | TC2-84 | KU737117 |
| *LFY* | *I. yunguiensis* | TC2-85 | KU737118 |
| *LFY* | *I. orientensis* | SY1-101 | KU737119 |
| *LFY* | *I. orientensis* | SY1-102 | KU737120 |
| *LFY* | *I. orientensis* | SY1-103 | KU737121 |
| *LFY* | *I. orientensis* | SY1-105 | KU737122 |
| *LFY* | *I. orientensis* | SY1-106 | KU737123 |
| *LFY* | *I. orientensis* | SY1-416 | KU737124 |
| *LFY* | *I. orientensis* | SY1-417 | KU737125 |
| *LFY* | *I. orientensis* | SY1-418 | KU737126 |
| *LFY* | *I. orientensis* | SY1-419 | KU737127 |
| *LFY* | *I. orientensis* | SY1-420 | KU737128 |
| *LFY* | *I. orientensis* | SY1-51 | KU737129 |
| *LFY* | *I. orientensis* | SY1-52 | KU737130 |
| *LFY* | *I. orientensis* | SY1-53 | KU737131 |
| *LFY* | *I. orientensis* | SY1-54 | KU737132 |
| *LFY* | *I. orientensis* | SY1-55 | KU737133 |
| *LFY* | *I. orientensis* | SY1-61 | KU737134 |
| *LFY* | *I. orientensis* | SY1-62 | KU737135 |
| *LFY* | *I. orientensis* | SY1-63 | KU737136 |
| *LFY* | *I. orientensis* | SY1-64 | KU737137 |
| *LFY* | *I. orientensis* | SY1-65 | KU737138 |
| *LFY* | *I. orientensis* | SY1-91 | KU737139 |
| *LFY* | *I. orientensis* | SY1-92 | KU737140 |
| *LFY* | *I. orientensis* | SY1-94 | KU737141 |
| *LFY* | *I. orientensis* | SY1-95 | KU737142 |
| *LFY* | *I. orientensis* | SY1-96 | KU737143 |
| *LFY* | *I. orientensis* | SY2-101 | KU737144 |
| *LFY* | *I. orientensis* | SY2-1011 | KU737145 |
| *LFY* | *I. orientensis* | SY2-1015 | KU737146 |
| *LFY* | *I. orientensis* | SY2-1016 | KU737147 |
| *LFY* | *I. orientensis* | SY2-107 | KU737148 |
| *LFY* | *I. orientensis* | SY2-34 | KU737149 |
| *LFY* | *I. orientensis* | SY2-35 | KU737150 |
| *LFY* | *I. orientensis* | SY2-36 | KU737151 |
| *LFY* | *I. orientensis* | SY2-37 | KU737152 |
| *LFY* | *I. orientensis* | SY2-38 | KU737153 |
| *LFY* | *I. orientensis* | SY2-41 | KU737154 |
| *LFY* | *I. orientensis* | SY2-42 | KU737155 |
| *LFY* | *I. orientensis* | SY2-43 | KU737156 |
| *LFY* | *I. orientensis* | SY2-46 | KU737157 |
| *LFY* | *I. orientensis* | SY2-47 | KU737158 |
| *LFY* | *I. orientensis* | SY2-71 | KU737159 |
| *LFY* | *I. orientensis* | SY2-72 | KU737160 |
| *LFY* | *I. orientensis* | SY2-74 | KU737161 |
| *LFY* | *I. orientensis* | SY2-75 | KU737162 |
| *LFY* | *I. orientensis* | SY2-76 | KU737163 |
| *LFY* | *I. orientensis* | SY2-81 | KU737164 |
| *LFY* | *I. orientensis* | SY2-82 | KU737165 |
| *LFY* | *I. orientensis* | SY2-83 | KU737166 |
| *LFY* | *I. orientensis* | SY2-84 | KU737167 |
| *LFY* | *I. orientensis* | SY2-87 | KU737168 |
| *LFY* | *I. sinensis* | HT101 | KU737169 |
| *LFY* | *I. sinensis* | HT102 | KU737170 |
| *LFY* | *I. sinensis* | HT103 | KU737171 |
| *LFY* | *I. sinensis* | HT104 | KU737172 |
| *LFY* | *I. sinensis* | HT106 | KU737173 |
| *LFY* | *I. sinensis* | HT1-51 | KU737174 |
| *LFY* | *I. sinensis* | HT1-52 | KU737175 |
| *LFY* | *I. sinensis* | HT1-53 | KU737176 |
| *LFY* | *I. sinensis* | HT1-54 | KU737177 |
| *LFY* | *I. sinensis* | HT1-55 | KU737178 |
| *LFY* | *I. sinensis* | HT1-71 | KU737179 |
| *LFY* | *I. sinensis* | HT1-73 | KU737180 |
| *LFY* | *I. sinensis* | HT1-74 | KU737181 |
| *LFY* | *I. sinensis* | HT1-75 | KU737182 |
| *LFY* | *I. sinensis* | HT1-76 | KU737183 |
| *LFY* | *I. sinensis* | HT1-83 | KU737184 |
| *LFY* | *I. sinensis* | HT1-85 | KU737185 |
| *LFY* | *I. sinensis* | HT1-87 | KU737186 |
| *LFY* | *I. sinensis* | HT1-88 | KU737187 |
| *LFY* | *I. sinensis* | HT1-89 | KU737188 |
| *LFY* | *I. sinensis* | HT1-913 | KU737189 |
| *LFY* | *I. sinensis* | HT1-915 | KU737190 |
| *LFY* | *I. sinensis* | HT1-917 | KU737191 |
| *LFY* | *I. sinensis* | HT1-93 | KU737192 |
| *LFY* | *I. sinensis* | HT1-94 | KU737193 |
| *LFY* | *I. sinensis* | JD1-214 | KU737194 |
| *LFY* | *I. sinensis* | JD1-22 | KU737195 |
| *LFY* | *I. sinensis* | JD1-23 | KU737196 |
| *LFY* | *I. sinensis* | JD1-24 | KU737197 |
| *LFY* | *I. sinensis* | JD1-26 | KU737198 |
| *LFY* | *I. sinensis* | JD1-43 | KU737199 |
| *LFY* | *I. sinensis* | JD1-44 | KU737200 |
| *LFY* | *I. sinensis* | JD1-45 | KU737201 |
| *LFY* | *I. sinensis* | JD1-46 | KU737202 |
| *LFY* | *I. sinensis* | JD1-47 | KU737203 |
| *LFY* | *I. sinensis* | JD1-51 | KU737204 |
| *LFY* | *I. sinensis* | JD1-52 | KU737205 |
| *LFY* | *I. sinensis* | JD1-53 | KU737206 |
| *LFY* | *I. sinensis* | JD1-55 | KU737207 |
| *LFY* | *I. sinensis* | JD1-57 | KU737208 |
| *LFY* | *I. sinensis* | JD1-61 | KU737209 |
| *LFY* | *I. sinensis* | JD1-63 | KU737210 |
| *LFY* | *I. sinensis* | JD1-64 | KU737211 |
| *LFY* | *I. sinensis* | JD1-65 | KU737212 |
| *LFY* | *I. sinensis* | JD1-66 | KU737213 |
| *LFY* | *I. sinensis* | JD1-81 | KU737214 |
| *LFY* | *I. sinensis* | JD1-83 | KU737215 |
| *LFY* | *I. sinensis* | JD1-84 | KU737216 |
| *LFY* | *I. sinensis* | JD1-86 | KU737217 |
| *LFY* | *I. sinensis* | JD1-88 | KU737218 |
| *LFY* | *I. sinensis* | JD2-11 | KU737219 |
| *LFY* | *I. sinensis* | JD2-12 | KU737220 |
| *LFY* | *I. sinensis* | JD2-13 | KU737221 |
| *LFY* | *I. sinensis* | JD2-14 | KU737222 |
| *LFY* | *I. sinensis* | JD2-15 | KU737223 |
| *LFY* | *I. sinensis* | JD2-421 | KU737224 |
| *LFY* | *I. sinensis* | JD2-422 | KU737225 |
| *LFY* | *I. sinensis* | JD2-424 | KU737226 |
| *LFY* | *I. sinensis* | JD2-425 | KU737227 |
| *LFY* | *I. sinensis* | JD2-427 | KU737228 |
| *LFY* | *I. sinensis* | JD2-4914 | KU737229 |
| *LFY* | *I. sinensis* | JD2-4915 | KU737230 |
| *LFY* | *I. sinensis* | JD2-492 | KU737231 |
| *LFY* | *I. sinensis* | JD2-494 | KU737232 |
| *LFY* | *I. sinensis* | JD2-496 | KU737233 |
| *LFY* | *I. sinensis* | JD2-51 | KU737234 |
| *LFY* | *I. sinensis* | JD2-52 | KU737235 |
| *LFY* | *I. sinensis* | JD2-54 | KU737236 |
| *LFY* | *I. sinensis* | JD2-57 | KU737237 |
| *LFY* | *I. sinensis* | JD2-59 | KU737238 |
| *LFY* | *I. sinensis* | JD2-81 | KU737239 |
| *LFY* | *I. sinensis* | JD2-82 | KU737240 |
| *LFY* | *I. sinensis* | JD2-83 | KU737241 |
| *LFY* | *I. sinensis* | JD2-84 | KU737242 |
| *LFY* | *I. sinensis* | JD2-85 | KU737243 |
| *LFY* | *I. sinensis* | NX1-215 | KU737244 |
| *LFY* | *I. sinensis* | NX1-216 | KU737245 |
| *LFY* | *I. sinensis* | NX1-217 | KU737246 |
| *LFY* | *I. sinensis* | NX1-22 | KU737247 |
| *LFY* | *I. sinensis* | NX1-220 | KU737248 |
| *LFY* | *I. sinensis* | NX1-315 | KU737249 |
| *LFY* | *I. sinensis* | NX1-319 | KU737250 |
| *LFY* | *I. sinensis* | NX1-320 | KU737251 |
| *LFY* | *I. sinensis* | NX1-511 | KU737252 |
| *LFY* | *I. sinensis* | NX1-54 | KU737253 |
| *LFY* | *I. sinensis* | NX1-57 | KU737254 |
| *LFY* | *I. sinensis* | NX1-58 | KU737255 |
| *LFY* | *I. sinensis* | NX1-59 | KU737256 |
| *LFY* | *I. sinensis* | NX1-71 | KU737257 |
| *LFY* | *I. sinensis* | NX1-712 | KU737258 |
| *LFY* | *I. sinensis* | NX1-714 | KU737259 |
| *LFY* | *I. sinensis* | NX1-73 | KU737260 |
| *LFY* | *I. sinensis* | NX1-78 | KU737261 |
| *LFY* | *I. sinensis* | NX1-91 | KU737262 |
| *LFY* | *I. sinensis* | NX1-92 | KU737263 |
| *LFY* | *I. sinensis* | NX1-95 | KU737264 |
| *LFY* | *I. sinensis* | NX1-96 | KU737265 |
| *LFY* | *I. sinensis* | NX1-97 | KU737266 |
| *LFY* | *I. sinensis* | NX31 | KU737267 |
| *LFY* | *I. sinensis* | NX34 | KU737268 |
| *LFY* | *I. sinensis* | TD1-55 | KU737269 |
| *LFY* | *I. sinensis* | TD1-57 | KU737270 |
| *LFY* | *I. sinensis* | TD1-72 | KU737271 |
| *LFY* | *I. sinensis* | TD1-74 | KU737272 |
| *LFY* | *I. sinensis* | TD1-75 | KU737273 |
| *LFY* | *I. sinensis* | TD1-76 | KU737274 |
| *LFY* | *I. sinensis* | TD1-77 | KU737275 |
| *LFY* | *I. sinensis* | TD31 | KU737276 |
| *LFY* | *I. sinensis* | TD32 | KU737277 |
| *LFY* | *I. sinensis* | TD33 | KU737278 |
| *LFY* | *I. sinensis* | TD34 | KU737279 |
| *LFY* | *I. sinensis* | TD35 | KU737280 |
| *LFY* | *I. sinensis* | TD51 | KU737281 |
| *LFY* | *I. sinensis* | TD53 | KU737282 |
| *LFY* | *I. sinensis* | TD56 | KU737283 |
| *LFY* | *I. sinensis* | TD81 | KU737284 |
| *LFY* | *I. sinensis* | TD82 | KU737285 |
| *LFY* | *I. sinensis* | TD84 | KU737286 |
| *LFY* | *I. sinensis* | TD85 | KU737287 |
| *LFY* | *I. sinensis* | TD86 | KU737288 |
| *LFY* | *I. sinensis* | TD91 | KU737289 |
| *LFY* | *I. sinensis* | TD92 | KU737290 |
| *LFY* | *I. sinensis* | TD94 | KU737291 |
| *LFY* | *I. sinensis* | TD95 | KU737292 |
| *LFY* | *I. sinensis* | TD96 | KU737293 |
| *LFY* | *I. sinensis* | TT1-31 | KU737294 |
| *LFY* | *I. sinensis* | TT1-311 | KU737295 |
| *LFY* | *I. sinensis* | TT1-317 | KU737296 |
| *LFY* | *I. sinensis* | TT1-32 | KU737297 |
| *LFY* | *I. sinensis* | TT1-37 | KU737298 |
| *LFY* | *I. sinensis* | TT1-510 | KU737299 |
| *LFY* | *I. sinensis* | TT1-52 | KU737300 |
| *LFY* | *I. sinensis* | TT1-53 | KU737301 |
| *LFY* | *I. sinensis* | TT1-55 | KU737302 |
| *LFY* | *I. sinensis* | TT1-57 | KU737303 |
| *LFY* | *I. sinensis* | TT1-72 | KU737304 |
| *LFY* | *I. sinensis* | TT1-73 | KU737305 |
| *LFY* | *I. sinensis* | TT1-75 | KU737306 |
| *LFY* | *I. sinensis* | TT1-76 | KU737307 |
| *LFY* | *I. sinensis* | TT1-77 | KU737308 |
| *LFY* | *I. sinensis* | TT1-82 | KU737309 |
| *LFY* | *I. sinensis* | TT1-83 | KU737310 |
| *LFY* | *I. sinensis* | TT1-84 | KU737311 |
| *LFY* | *I. sinensis* | TT1-85 | KU737312 |
| *LFY* | *I. sinensis* | TT1-87 | KU737313 |
| *LFY* | *I. sinensis* | TT1-910 | KU737314 |
| *LFY* | *I. sinensis* | TT1-92 | KU737315 |
| *LFY* | *I. sinensis* | TT1-94 | KU737316 |
| *LFY* | *I. sinensis* | TT1-95 | KU737317 |
| *LFY* | *I. sinensis* | TT1-99 | KU737318 |
| *LFY* | *I. sinensis* | XN106 | KU737319 |
| *LFY* | *I. sinensis* | XN11 | KU737320 |
| *LFY* | *I. sinensis* | XN1-101 | KU737321 |
| *LFY* | *I. sinensis* | XN1-102 | KU737322 |
| *LFY* | *I. sinensis* | XN1-104 | KU737323 |
| *LFY* | *I. sinensis* | XN1-105 | KU737324 |
| *LFY* | *I. sinensis* | XN12 | KU737325 |
| *LFY* | *I. sinensis* | XN14 | KU737326 |
| *LFY* | *I. sinensis* | XN1-41 | KU737327 |
| *LFY* | *I. sinensis* | XN1-417 | KU737328 |
| *LFY* | *I. sinensis* | XN1-45 | KU737329 |
| *LFY* | *I. sinensis* | XN1-49 | KU737330 |
| *LFY* | *I. sinensis* | XN15 | KU737331 |
| *LFY* | *I. sinensis* | XN16 | KU737332 |
| *LFY* | *I. sinensis* | XN1-91 | KU737333 |
| *LFY* | *I. sinensis* | XN1-911 | KU737334 |
| *LFY* | *I. sinensis* | XN1-915 | KU737335 |
| *LFY* | *I. sinensis* | XN1-92 | KU737336 |
| *LFY* | *I. sinensis* | XN1-920 | KU737337 |
| *LFY* | *I. sinensis* | XN438 | KU737338 |
| *LFY* | *I. sinensis* | XN71 | KU737339 |
| *LFY* | *I. sinensis* | XN73 | KU737340 |
| *LFY* | *I. sinensis* | XN74 | KU737341 |
| *LFY* | *I. sinensis* | XN75 | KU737342 |
| *LFY* | *I. sinensis* | XN76 | KU737343 |
| *LFY* | *I. hypsophila* | BY | KU745461 |
