# Supplementary material for: The speciation and adaptation of the polyploids: a case study of the Chinese Isoetes L. diploid-polyploid complex
Source: BMC Evol Biol. 2020 Sep 14;20:118. doi: 10.1186/s12862-020-01687-4 (PMC7490897; doi:10.1186/s12862-020-01687-4)
Supplement: Supplementary file 1 — Additional file 1: Table S1. The serial numbers of plastid DNA sequences in this study. Table S2. The serial numbers of nuclear DNA sequences in this study. Table S3. Haplotypes information of nuclear DNA data. Table S4. Haplotypes information of cpDNA data. Table S5. Location records used for ecological niche modeling. Table S6. Results of the nonparametric Kruskal test applied for the populations whose maternal contributor are different in the allopolyploid populations of I.sinensis. [file 12862_2020_1687_MOESM1_ESM.zip › Table S5.docx]

Table S5. Localities used for ecological niche modeling.

| Species | Longitude | | Latitude | |
| --- | --- | --- | --- | --- |
| *I. yunguiensis* | 98.583 | 25.003 | |  |
|  | 98.566 | 24.822 | |  |
|  | 106.399 | 26.499 | |  |
|  | 106.283 | 26.429 | |  |
|  | 105.385 | 26.737 | |  |
|  | 105.271 | 26.692 | |  |
|  | 105.387 | 26.733 | |  |
| *I. sinensis* | 109.617 | 26.750 | |  |
|  | 119.233 | 29.467 | |  |
|  | 119.250 | 29.467 | |  |
|  | 112.283 | 28.183 | |  |
|  | 118.150 | 29.700 | |  |
| *I. taiwanensis* | 121.546 | 25.166 | |  |
|  | 121.557 | 25.177 | |  |
|  | 121.549 | 25.162 | |  |
|  | 121.550 | 25.167 | |  |
|  | 121.571 | 24.923 | |  |
|  | 118.383 | 24.450 | |  |
