# Supplementary material for: The speciation and adaptation of the polyploids: a case study of the Chinese Isoetes L. diploid-polyploid complex
Source: BMC Evol Biol. 2020 Sep 14;20:118. doi: 10.1186/s12862-020-01687-4 (PMC7490897; doi:10.1186/s12862-020-01687-4)
Supplement: Supplementary file 2 — Additional file 2: Figure S1. Kernel density plots of the six environmental variables for the populations whose maternal contributor are different in the allopolyploid of I. sinensis. I. sinensis (tai) indicates the maternal contributor of the population is I. taiwanensis and I. sinensis (yun) means the maternal contributor of the population is I. yunguiensis. Differentiation between different populations and the results of the nonparametric Kruskal-test are indicated in each plot. The equal sign indicates the lack of significant differences (p ≥ 0.05), while the significant differences are indicated by either higher or lower sign (p < 0.05). [file 12862_2020_1687_MOESM2_ESM.pdf]

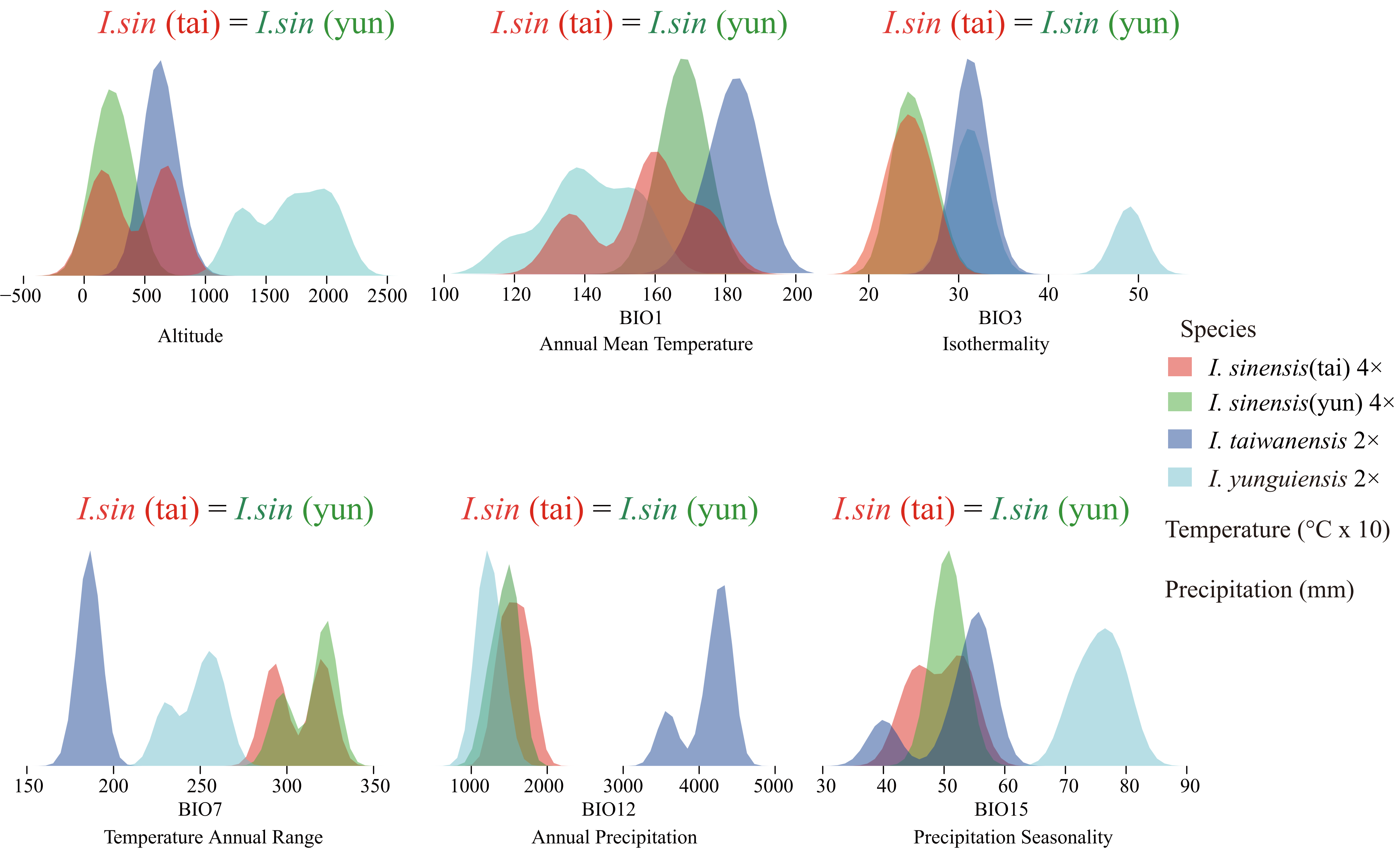
I.sin (tai) = I.sin (yun)
−500
0
500
1000
1500
2000
2500
Altitude
I.sin (tai) = I.sin (yun)
100
120
140
160
180
200
BIO1
Annual Mean Temperature
I.sin (tai) = I.sin (yun)
20
30
40
50
BIO3
Isothermality
Species
I. sinensis(tai) 4×
I. sinensis(yun) 4×
I. taiwanensis 2×
I. yunguiensis 2×
Temperature (°C x 10)
Precipitation (mm)
I.sin (tai) = I.sin (yun)
150
200
250
300
350
BIO7
Temperature Annual Range
I.sin (tai) = I.sin (yun)
1000
2000
3000
4000
5000
BIO12
Annual Precipitation
I.sin (tai) = I.sin (yun)
30
40
50
60
70
80
90
BIO15
Precipitation Seasonality
